# Defining the antigen receptor-dependent regulatory network that induces arrest of cycling immature B-lymphocytes

**DOI:** 10.1186/1752-0509-4-169

**Published:** 2010-12-09

**Authors:** Mohammad Sarwar Jamal, Srikanth Ravichandran, Noor Jailkhani, Samrat Chatterjee, Raina Dua, Kanury VS Rao

**Affiliations:** 1 Immunology Group, International Centre for Genetic Engineering and Biotechnology, Aruna Asaf Ali Marg, New Delhi - 110067, India; 2 Department of Physics and Astrophysics, University of Delhi, New Delhi - 110007, India

## Abstract

**Background:**

Engagement of the antigen receptor on immature B-lymphocytes leads to cell cycle arrest, and subsequent apoptosis. This is an essential process for eliminating self reactive B cells during its different stages of development. However, the mechanism by which it is achieved is not completely understood.

**Results:**

Here we employed a systems biology approach that combined extensive experimentation with *in silico *methodologies to chart the network of receptor-activated pathways that mediated the arrest of immature B cells in the G1 phase of the cell cycle. Interestingly, we found that only a sparse network of signaling intermediates was recruited upon engagement of the antigen receptor. This then led to the activation of a restricted subset of transcription factors, with the consequent induction of genes primarily involved in the cell death pathway. Subsequent experiments revealed that the weak initiation of intracellular signaling pathways derived from desensitization of the receptor-proximal protein tyrosine kinase Lyn, to receptor-dependent activation. Intriguingly, the desensitization was a result of the constitutive activation of this kinase in unstimulated cells, which was likely maintained through a regulatory feedback loop involving the p38 MAP kinase. The high basal activity then attenuated the ability of the antigen receptor to recruit Lyn, and thereby also the downstream signaling intermediates. Finally, integration of these results into a mathematical model provided further substantiation to the novel finding that the ground state of the intracellular signaling machinery constitutes an important determinant of the outcome of receptor-induced cellular responses.

**Conclusions:**

Our results identify the global events leading to the G1 arrest and subsequent apoptosis in immature B cells upon receptor activation.

## Background

Cellular responses to environmental cues are mediated through activation of the signal transduction machinery. This machinery is best represented as a complex network that, in turn, governs the decision-making capabilities of the cell [1,2]. Engagement of a cell surface receptor induces activation of signal transduction cascades that involve a series of phosphorylation/dephosphorylation events. These phosphorylation-dependent signaling events eventually transduce signal to transcription factors (TFs), with the latter then modulating expression levels of the downstream genes [3]. The cellular response thus elicited is a consequence of this alteration in the gene expression profile. Information processing is an integral part of signal transmission wherein calibration of both quantitative and qualitative features of the signal is facilitated by the many regulatory elements, or motifs, that are distributed across the signal transduction and transcription regulatory networks [4]. These regulatory elements constitute emergent features of the corresponding networks and they play a critical role in ensuring that the cellular phenotypic response is contextually derived from the nature of the inducing stimulus [5].

Several studies have at least partially delineated the emergent features of the signaling network that are generated in response to engagement of a variety of cell surface receptors [6-8]. Similarly, topological alterations in the transcription regulatory network that are generated under specific conditions of cell activation have also been mapped [9]. However, a more global perspective that rationalizes how these two networks integrate to ensure context-specificity of the cellular response is presently lacking. An understanding at this level, however, is critical for eventual resolution of the mechanisms that underlie cell fate decisions, as well as those that lead to aberrations in cellular behavior [10].

In the present study we adopted a systems biology approach to address this question. For this we took the murine B-lymphoma cell line CH1 as the model system. These cells are a prototype of the transitional stage of immature B cells and previous studies have shown that stimulation of these cells through the B-cell antigen receptor (BCR) leads to late G1 arrest, which is then followed by apoptosis [11,12]. This response to BCR activation is also reminiscent of that seen for immature B cells *in vivo*, and contributes towards the elimination of self-reactive cells from the peripheral B-cell repertoire [13]. It was therefore of interest to delineate the regulatory network involved in transmission of receptor-activated signals, to eventually enforce the cell cycle arrest response.

A combination of experimental with *in silico *approaches enabled us to map the network of pathways emanating from the BCR, and leading up to the induction of genes responsible for the G1 arrest. A detailed analysis of the time-dependent phosphorylation of several signaling intermediates revealed that BCR-engagement resulted in only a partial and transient activation of the signaling network. A direct consequence of this was a weak perturbation of the transcription regulatory network, which in turn led to the expression of only those genes that were involved in the cell death pathway. These latter findings were facilitated through a large-scale survey of TFs for their sensitivities to BCR-activation, and by a microarray analysis of the gene expression profile in stimulated cells followed by experimental verification of the functional roles of the early induced genes. Interestingly, our subsequent experiments revealed that integration between the signaling and the transcription regulatory networks was controlled by the MAP kinase signaling intermediate p38. This control was enforced through a receptor-associated phosphatase and involved the feedback regulation of Lyn, the kinase that initiates signaling from the BCR. It was this feedback control exercised at the level of signal initiation that then eventually resulted in the expression of genes causing cell cycle arrest. An incorporation of these observations into a mathematical model provided further insights into how changes in the basal activation state of the early intermediates defines sensitivity of the signaling machinery to a given cell surface receptor. Thus, our studies also reveal the etiology of cell type-specific responses to a given stimulus.

## Methods

### Cell Culture, Stimulation and detection of phosphoproteins

The experimental conditions employed in this study were first established in standardization experiments involving both different doses of anti-IgM, and variations in the stimulation times. A saturating effect on G1 arrest of CH1 cells was seen at an anti-IgM concentration of between 3-5 μg/ml, with no additional effect also when the stimulation time was extended beyond 1 h. Consequently, stimulation of CH1 cells for 1 h with a final anti-IgM concentration of 5 μg/ml was taken as the optimal condition for our study. Consequently, CH1 cells were maintained at a density of 0.5 x10^6 ^cells/ml in RPMI 1640 supplemented with 10% fetal calf serum and 1X penicillin/streptomycin. They were stimulated with the F(ab)2 fragment of rabbit anti-mouse IgM (5 μg/ml) in RPMI for a period of up to 1 hr. At appropriate times thereafter, aliquots of cells were collected, centrifuged, and the cell pellets stored in liquid nitrogen. Just prior to electrophoresis, cells were lysed in lysis buffer (20 mM HEPES, 10 mM NaCl, 1.5 nM MgCl2, 0.2 mM EDTA, 0.5% Triton X-100, 0.5 mM DTT, 1 mM sodium orthovanadate, 1 mM NaF, and a cocktail of protease inhibitors) followed by removal of the nuclear material and other debris through centrifugation. The detergent-soluble proteins were then resolved by SDS-PAGE. Specific proteins and phosphoproteins (all phospho-specific antibodies, as described in Additional File 1: Supplemental Table S6, were from Cell Signaling Technologies) were detected by Western blot using appropriate antibodies. For this, lysates were resolved by SDS-PAGE and then transferred to a nitrocellulose membrane (Hybond ECL; Amersham Biosciences). The membrane was incubated in odyssey blocking buffer for 2 h with gentle shaking at 37°C. The blocking buffer was replaced with an appropriate dilution of primary antibody in odyssey buffer with 10% PBS and incubation was continued at 4°C over night with gentle shaking. Thereafter, the blots were washed thrice with PBST for 5 min each. After washing, the blots were incubated with infrared dye labeled secondary antibodies (1:15,000 dilutions, obtained from Licor Inc, USA) at 37°C for 2 h. Blots were scanned using Odyssey scanner using an 800 nm laser, and band intensities were determined by using Odyssey software. Minimum intensity surrounding the bands on the film was taken as its background and subtracted to give the true intensity. All blots were re-probed for GAPDH as loading controls. Intensities were normalized against the intensities of GAPDH molecule.

### Co-Immunoprecipitation and Western blot analysis

Lysates were prepared from between 2-5 × 10^7 ^cells in a buffer containing 20 mM Tris-HCl, pH 7.5, 150 mM NaCl, 1 mM EDTA, 1 mM EGTA, 1% Triton X-100, and a phosphatase inhibitor cocktail (Roche, Mannheim, Germany). The clarified supernatant was quantified for protein concentration by the Bradford assay (Bio-Rad Laboratories). For immunoprecipitation, 1 mg of total protein in 500 μl of lysis buffer was incubated with 20 μl of Sepharose 4B (Amersham) for 1 h at 4°C. The pre-cleared lysate was then incubated with 2 μg of the antibody overnight at 4°C, followed by 20 μl of protein A-agarose beads for 2 h at 4°C. After five washings in lysis buffer, the beads were boiled in Laemmli buffer, and the proteins were separated by SDS-PAGE. Further steps for Western blot analyses were as described above.

### In-vitro Phosphatase Assay

This was done essentially as described earlier, using para-nitrophenyl phosphate as the substrate [14]. That comparable amounts of the phosphatase SHP-1 were present in samples from the individual groups was again ensured through Western blot analyses on parallel sets of immunoprecipitates.

### Cell cycle analysis by flow cytometry

Cells were plated in the wells of a 96-well plate and stimulated with anti-IgM for 1 h after which the cells were washed and replaced with fresh medium without anti-IgM. At 16 h after initiation of stimulation, the cells were harvested and stained with propidium iodide for analysis by flow cytometry. The extent of cell cycle arrest was determined by measuring the relative proportion of cells in the G0/G1, versus the S and G2/M phases in each of the experimental groups.

### siRNA-mediated suppression of BCR-induced genes

All the specific siRNAs were procured from Qiagen. HiPerfect (Qiagen) was used for transfection of cells with the siRNAs (at a final concentration of 100 nM) strictly following the protocol supplied by the manufacturer. In initial standardization experiments, the silencing obtained was between 70 and 95% at 48 h after transfection, as detected by RT-PCR. The list of catalog numbers and source of siRNAs used is provided in Additional File 1: Supplemental Table S5. For all of the experiments described here, a parallel control set was always included wherein cells were treated with siRNA specific for GFP (mock siRNA-treatment). After 48 hr of siRNA transfection cells were stimulated for 1 h and at the16 h time point they were harvested and stained with propidium iodide for acquisition and subsequent cell cycle analysis (Additional File 1: Supplemental Fig. S2). In experiments involving the use of specific inhibitors, these inhibitors were added to cells at 30 min prior to stimulation. At the end of the 1 h stimulation period with anti-IgM, however, these inhibitors were also washed out and no fresh inhibitor was added for the remainder of the experiment.

### RNA Isolation and Realtime-PCR

Total RNA was isolated with TRIzol (Invitrogen) and digested with RNase free DNase I prior to the reverse transcription reaction. Estimation of relative transcript levels by real time PCR was obtained as a commercial service from Labindia Life Sciences (Gurgaon, India). The assay and analysis were performed as previously described [15]. Also refer additional file 1 for detailed methods.

### Confocal Microscopy

#### Staining Protocol

Staining was performed as described [16]. To examine co-localization between p38 and SHP-1, CH1 cells (0.7 million) seeded on glass coverslip coated with CellTak (BD Bioscience) were stimulated with anti-IgM for 5'min. Cells were fixed with 3% paraformaldehyde in PBS for 10 min at room temperature followed by quenching with 50 mM ammonium chloride for 10 min. Fixed cells were permeabilized by incubating with 0.2% Triton X-100 in PBS for 5 minutes followed by blocking (3% bovine serum albumin and 0.5% Tween20 in PBS) for 2 hours. Cells were incubated for 1-hour with respective primary antibodies (p38 and SHP-1 raised in mouse and rabbit respectively and diluted 1:100 with blocking buffer). This was followed by three washes with PBST and incubation with respective secondary antibodies (Alexa-Fluor 488 and Alexa-Flour 568 conjugated to anti-rabbit and anti-mouse IgG (Molecular Probes)). All cover slips were mounted on slides with Antifade (Biorad).

#### Image Capturing

Stained cells were observed with a Nikon TE 2000E laser scanning confocal microscope equipped with 60X/1.4 NA planapochromat DIC objective lens. Alexa Flour 488 and 568 were excited at 488 and 568 nm with an argon ion and He-Neon laser respectively. The emissions were recorded through emission filter set 515/30; 605/75. Serial confocal sections (0.5 μm thick) within a z-stack spanning a total thickness of 10-12 μm were taken in individual green and red channels using the motor drive focusing system. Images were acquired, with a scanning mode format of 512 × 512 pixels. The transmission and detector gains were set to achieve best signal to noise ratios and the laser powers were tuned to limit bleaching of fluorescence. The refractive index of the immersion oil used was 1.515 (Nikon). All settings were rigorously maintained for all experiments.

#### Image Analysis

All images were quantified using Image-Pro^® ^Plus version 6.0, a commercially available software package from Media Cybernetics.

The merged confocal images were subjected to co-localization analysis to determine the "Pearson Coefficient" proposed by [17].

$$\begin{array}{l} R=\frac{\sum \left(S1_{\text{i}}-S1_{\text{avg}})\times (S2_{\text{i}}-S2_{\text{avg}}\right)}{\sqrt{\sum {\left(S1_{\text{i}}-S1_{\text{avg}}\right)}^{2}\times \sum {\left(S2_{\text{i}}-S2_{\text{avg}}\right)}^{2}}} \end{array}$$

Where S1**_i _**is signal intensity of the ith pixels in channel 1; S1**_avg _**is the average intensity of all pixels in channel 1; S2**_i _**is signal intensity of the ith pixels in channel 2; S2**_avg _**is the average intensity of all pixels in channel 2.

About 50 cells were analyzed in 3 sets of slides for the co-localization studies. All the images are in the Tiff RGB 24 format. To reduce the unwanted background noise generated by the photomultiplier signal amplification, all the image stacks were treated with two-dimensional filters (Gaussian and sharpening filtering).

#### Protein/DNA arrays

Aliquots of either unstimulated cells, or cells stimulated with anti-IgM for the indicated times, were collected, centrifuged, and nuclear extracts were prepared as prescribed by the manufacturer. 10 μg of each nuclear extract was separately incubated with the biotinylated probe mix from the array kit for 30 min at 15°C. This mix contains oligonucleotides representing the consensus binding sites for 345 TFs. At the end of this incubation period, probes bound to transcription factors present in the nuclear extract were isolated by column chromatography, and these bound probes were then dissociated from the respective transcription factors by using the protocol recommended by the manufacturer. These samples were then hybridized (42°C for 16 h) with the Panomics Protein-DNA Spin Combo Array Kit (catalog number MA1215, http://www.panomics.com/index.php?id=product_18#product_listings_18) membranes, which contains an array of oligonucleotide sequences that are complementary to those of the TF binding sites in the probe mix (A complete list of target Cis-regulatory elements present in the array, their description and the corresponding references are provided by the manufacturer at http://www.panomics.com/index.php?id=product_18). The array was then washed, blocked, incubated with Steptavidin-HRP, and visualized by enhanced chemiluminesence. The blot was imaged using a PhosphoImager (Typhhoon 9210, GE Healthcare) and spot intensities were quantified using Imagequant TL. The quantified spots values were normalized against the average value of all the controls spotted on the border of membrane. Array experiment results from samples that were stimulated with anti-IgM were directly compared to the unstimulated control blot and spots that had increased by greater than 2-fold in the stimulation experiments were scored as positive for activation. All transcription factor array experiments were done in duplicate and only those TFs that were activated in both experiments were scored as positive for activation. Values for individual spot intensities are provided in Additional File 1: Supplemental Table S1, whereas the raw images of the blots are shown in Additional File 1: Supplemental Fig. S3

#### Identification of overrepresented Transcription Factor binding site for the set of early induced genes

The TRANSFAC^® ^database was used for our analysis and the commercial license for the same was obtained from BIOBASE. We employed the MATCH algorithm to identify the overrepresented transcription factor binding site in our gene of interests. TFBS was scanned for 1000 bp upstream and 500 bp downstream for the gene of interest. The gene sequence was for mouse was downloaded from *Genome browser *http://genome.ucsc.edu/cgi-bin/hgGateway?hgsid=145319238&clade=mammal&org=Mouse&db=mm8.

## Results

### BCR-dependent signaling arrests cycling of CH1 cells

**Figure 1:**
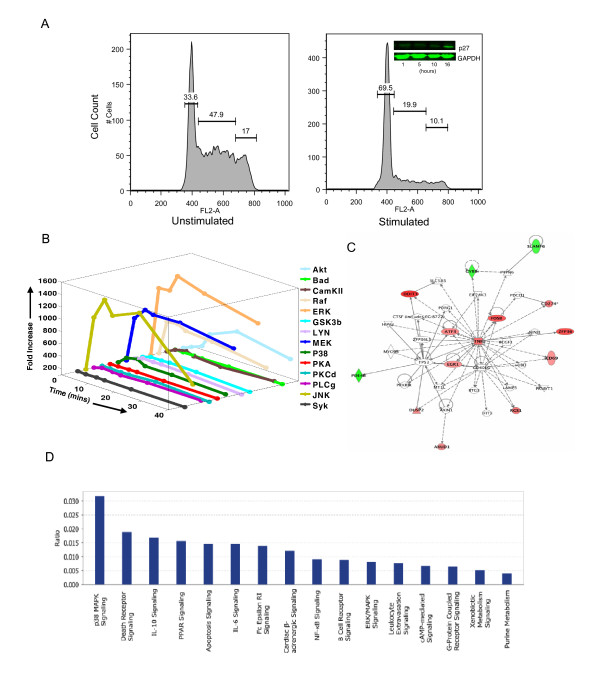
**Anti-IgM-dependent stimulation of CH1 cells leads to a poor activation of the signaling responses and induces cell cycle arrest**. Panel A shows the histograms representing the distribution of cells in each phase of the cell cycle observed by FACS analysis performed 16 hours after a 1 h stimulation of CH1 cells with anti-IgM. An arrest in cell cycle is clearly evident here, with a greater than 2-fold increase in the G1 phase population. Also shown in the inset is the result of a Western blot analysis revealing accumulation of p27 upon anti-IgM stimulation over the course of time. Panel B depicts the activation profiles of fourteen signaling intermediates probed by Western blots (see text for details and Additional File 1: Supplemental Fig. S1A) after anti-IgM stimulation. The plot represents mean value of quantified, normalized fold change in phosphorylation of the signaling intermediates in a time dependant manner obtained from three individual replicates and the over all S.D observed was < 10%. Also refer Additional File 1: Supplemental Fig. S1B for a detailed plot of the figure. An Ingenuity Pathway Analysis (IPA) of the set of early induced genes upon anti-IgM stimulation of CH1 cells is shown in Panel C. This network depicts the core module identified by IPA based on the list of early-induced genes used as the seed nodes. Here, the nodes in red represent the over-expressed genes while those in green are those whose expression was found to suppressed (Detailed key in Additional file 1). The canonical pathways identified by IPA for the set of early induced genes in CH1 cells and their corresponding significant levels of contribution by the early induced genes is shown in Panel D.

The murine B lymphoma CH1 cells express surface antigen receptors (BCR) of the IgM class. Transient stimulation of cell through these receptors with anti-IgM antibodies for 1 h resulted in an arrest of these cells in the G1 phase of the cell cycle. This arrest could be detected at 16 hr, with consequent apoptosis of the cells at the later time points (Figure 1A). Further, as expected [18], this G1 phase arrest was also characterized by an increase in intracellular levels of the p27 protein (Figure 1A). This protein inhibits the cell cycle-regulatory kinases CDK4/6 and CDK2 in a stoichiometric manner, thereby attenuating their ability to promote G1 to S phase transition [18]. Thus CH1 cells mimic primary immature B cells insofar as their response to BCR cross-linking and, therefore, provide a good model for studying antigen-induced clonal deletion of transitional stage B-lymphocytes [19].

We next examined the early signaling events activated by this receptor. For this cells were stimulated with anti-IgM and the time-dependent phosphorylation profiles of a panel of twenty signaling intermediates were examined by Western blot analyses. These signaling intermediates were selected on the basis that they collectively represented a diverse set of known canonical signaling pathways (References mentioned in Additional File 1: Supplemental Table S1). However, of the twenty molecules examined, we could observe BCR-dependent phosphorylation for only fourteen intermediates, with no significant effects being evident for the remaining six molecules (Data not shown). The remaining fourteen molecules were phosphorylated in a time-dependent manner by anti-IgM, although the individual profiles varied significantly (Additional File 1: Supplemental Fig. S1). This is evident from the quantified representations shown in Figure 1B. Thus stimulation of cells with anti-IgM resulted in vigorous phosphorylation of the members of the MAP kinase (MAPK) family ERK-1/2 (ERK) and JNK and, to a slightly lesser extent, also Raf-1 and MEK-1/2 (MEK) (Figure 1B). BCR-dependent phosphorylation, but with distinct kinetics and amplitude was also observed for Akt, PKCδ, p38, Lyn, and CaMKII (Figure 1B). Particularly surprisingly, however, was that stimulation elicited only a nominal phosphorylation response from the remaining intermediates, with molecules such as Syk, Bad, Gsk3β, PLCγ PKCδ and PKA achieving peak levels that were less than 2-fold above their respective basal values (Figure 1B). Thus, even this limited examination of a small panel of signaling intermediates highlights the sparse character of the BCR signaling network - with only a few signaling pathways being activated - in CH1 cells.

### BCR-dependent stimulation of CH1 cells induces the expression of cell cycle regulatory genes

We had previously examined induction of the early response genes in CH1 cells following stimulation with anti-IgM for 1 h [20]. A microarray analysis had identified that 19 genes were reproducibly upregulated to levels that were >2-fold above their basal value, whereas four genes were significantly downregulated [20](Accession No. E-MTAB-82). An Ingenuity Pathway Analysis using these twenty-three genes as the seed nodes yielded a top network, containing activities related to cell death and cancer, that incorporated 14 of these genes (11 upregulated and 3 downregulated) (Figure 1C). The canonical pathways affected by the nodes of this network are shown in Figure 1D. It is interesting to note that, in addition to the p38 pathway, the prominent pathways identified here were those that induced either cell death (death receptor signaling, apoptosis signaling), or anti-proliferative responses (IL-10 signaling, PPAR signaling, IL-6 signaling). For the sake of simplicity however we subsequently concentrated on only those eleven genes from this subset, whose expression levels were upregulated on stimulation of cells with anti-IgM (Figure 1C). The cellular functions attributed to the products of these genes include regulation of cell proliferation (ZFP36, CD69), regulation/repression of transcription (DDIT3, ATF3, EGR1, FOSB), inhibition of signal transduction (RGS1), and regulation of apoptosis/cell death (AXUD1, TNF).

### BCR-dependent regulation of transcription factor activities

The modulation of gene expression effected by signals emanating from a cell surface receptor is mediated through the regulation of transcription factor activities. Therefore, we next probed for the effects of anti-IgM stimulation on the activation of transcription factors (TFs). For these experiments we employed a commercial array in which oligonucleotides corresponding to the binding sites of 345 transcription factors were spotted. This array, therefore, enabled us to simultaneously assay the activation of a large subset of TFs.

**Figure 2:**
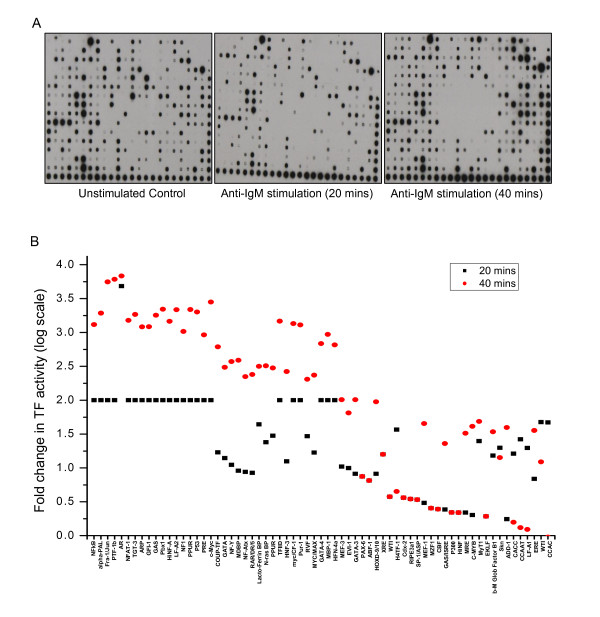
**BCR-dependent modulation of TF activities**. Panel A shows the representative arrays of transcription factor activation in CH1 cells in response to anti-IgM. Cells were stimulated with anti-IgM and the activation profiles of transcription factors probed by TF array either in unstimulated cells, or in cells stimulated for either 20 or 40 min with anti-IgM. Where, the unstimulated cells were treated as control. Plot in the panel B shows the quantified changes observed in the TF activity upon stimulation of CH1 cells with anti-IgM. Only the subset of TFs that showed >2 fold activation or repression in activity in either of the time points were plotted in log_10 _scale after. The nomenclature used to denote the TFs are the aliases provided by the array manufacturer, their corresponding Entrez Gene IDs are listed in Additional File 2.

Given that 1 h stimulation was sufficient to eventually induce G1 arrest, we measured the extent of TF activation in cells that were stimulated with anti-IgM for either 20 or 40 min and the representative blots thus obtained are shown in Figure 2A. A quantitative analysis of the intensities of the spots for each TF under the various conditions then yielded an anti-IgM-specific activation profile for the individual TFs. For our analysis, however, we only considered those TFs that were affected by >2-fold from their basal value to be either activated or inactivated in a BCR-dependent manner. Consistent with relatively poor activation of the signaling machinery observed earlier, anti-IgM-mediated stimulation also resulted in a weak perturbation of the TF network. Thus, of the 345 TFs examined activities of 279 remained unaffected, whereas that of 30 was suppressed. Further, although the remaining 36 TFs were activated by anti-IgM they - however - showed delayed kinetics with activation being detected only at 40 min of stimulation, (Figure 2B). Examples of these included NFKB1, FOSL1, PTFB1, NF1, and TRP53. In contrast, TF inactivation was relatively more rapid and was detectable by 20 min of stimulation in most cases. Examples of this latter group were GATA4, PAX6, Sp1, EP300, CMYB, NFATC2, and MZF1 (Figure 2B). The list of molecules shown in Figure 2B along with their corresponding Human Entrez Gene IDs is given in Additional File 2.

The activation profiles for a representative subset of the transcription factors probed here could be independently verified in Western blot experiments that monitored their increase in the nuclear compartment (Additional File 1: Supplemental Fig. S3B). However, there were some minor differences that could be observed in the TF activation pattern in the case of p-p53 and cMyc (Additional File 1: Supplemental Fig. S3B). Overall this validation supports that the results in Figure 2 indeed identify the BCR-sensitive TFs in CH1 cells. Further, at least some of these TFs may be expected to be involved in driving the arrest of actively cycling cells in the G1 phase.

### Defining the key transcriptional regulators that enforce the cellular response

**Figure 3:**
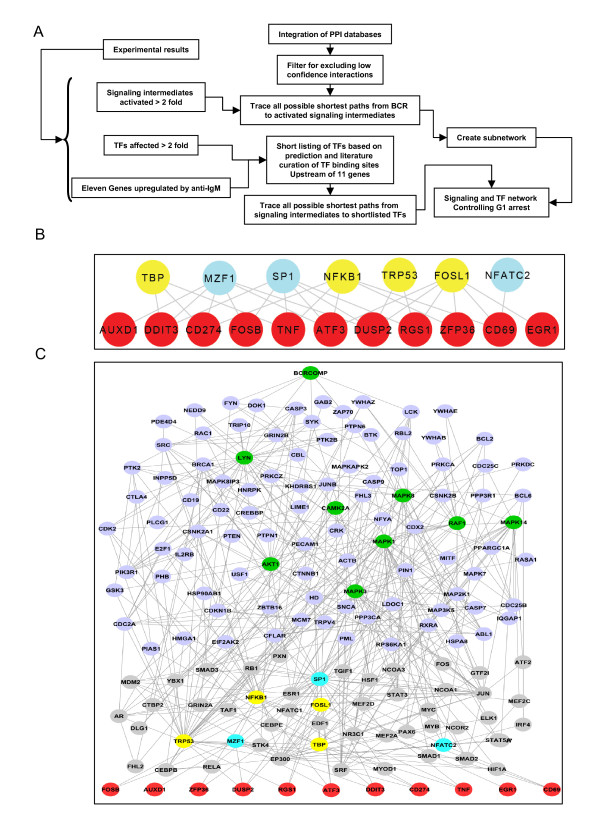
**Extracting the BCR-dependent network that regulates the cellular response**. The Schematic of the analysis performed to identify the core regulatory network inducing G1 arrest upon anti-IgM stimulation by combining our experimental data and publicly available PPI databases using network analysis is shown in panel A. Panel B depicts the dense overlapping regulatory module identified for TFs regulating the early induced genes due to BCR receptor stimulus. This was achieved by applying TFBS prediction algorithm and literature curation. The red nodes are the early genes induced genes. The yellow nodes represent TFs activated while the blue nodes represent the TFs repressed either at 20 or 40 minutes. Panel C shows the network emanating from BCR that induces changes in the activity of key signaling intermediates analyzed (green nodes) leading to either activation or repression of the set of transcription factors identified from TF array (The nodes are colour coded as in panel B). The nodes in light blue and grey represent other signaling and transcriptional intermediates identified from the network analysis. The set of genes affected due to anti-IgM stimulation are represented as red nodes. All the intermediate nodes are labeled to human orthologs with their corresponding Entrez Gene ID.

Our cumulative experiments so far helped to describe at least some of the signaling events activated by the BCR, as well as their downstream effects on TF activation, and the consequent gene expression. It was, therefore, of interest to synthesize these data to generate a more integrated perspective on the BCR-regulated arrest of cycling CH1 cells. To do this we combined our experimental results with an *in silico *approach as illustrated in Figure 3A. Our goal here was to extract the regulatory network that could be implicated in this process.

As the first step, we sought to identify those TFs that could be involved in regulating the set of early-induced genes described in Figure 1C. For this we employed the MATCH software to scan for TF binding sites that were over-represented in the promoter regions (i.e. from -1000 nt to +500 nt relative to the transcription start site) of each of the eleven early-induced genes. In addition, we also surveyed the literature for TFs that have been experimentally demonstrated to regulate expression of these target genes. Results from both approaches were then combined to yield a list of sixteen TFs for these eleven genes (see Additional File 1: Supplemental Table S4). From this list we next selected those TFs that were also either activated or suppressed by anti-IgM in Figure 2. This exercise resulted in the further short-listing of seven of these TFs. Importantly, the identification of several of these was also supported by experimental evidence in the literature demonstrating their roles in regulating expression of at least some of the target genes (Additional File 1: Supplemental Table S4). Linking these seven TFs to the target genes then yielded a dense overlapping regulatory (DOR) network as shown in Figure 3B. Such a DOR network represents a typical regulatory module that is expected for the regulation of multiple genes by a common set of TFs [21,22].

Of the seven TFs described in Figure 3B, TBP, NFKB1, TRP53, and FOSL1 were all activated upon stimulation of cells with anti-IgM (Figure 2). Of these TBP is a component of the general transcription factor TFIID [23], while both NFKB1 and TRP53 are known regulators of gene expression [24-28]. Finally, FOSL1 is an oncogene product with a role in tumor formation [29]. Activity of the remaining three TFs - MZF1, Sp1, and NFATc2 - was, however, suppressed in response to BCR stimulation. Here MZF1 is known for its regulation of apoptosis [30], whereas NAFTc2 and Sp1 can both act as repressors of gene expression in specific instances [31,32]. Thus the BCR-dependent activation profile of these seven TFs appears to be consistent with the induced expression of the early response genes through the links described in Figure 3B.

### Construction of an in silico network that links BCR-signaling to gene expression

To extract the network of pathways linking BCR activation to the cellular response, we first merged the BIND[33], DIP[34], IntAct [35], MINT [36], Human Protein Reference Database [37] and Protein-Protein interaction database [38] PPI databases to generate a compilation of all known reported PPIs. Eliminating those interactions that lacked experimental support from at least two independent studies then refined the resulting network. This resulted in a core undirected network of about 4300 nodes and 10700 edges. Here the CD79α and CD79β subunits associated with the BCR [39] were taken together and considered as a single BCR complex.

Shortest path analysis of networks is generally considered to represent a reliable method for capturing information on the transduction of signals through the various intermediate nodes [40]. Further, our experiments in Figure 1B had also helped to distinguish at least some of the signaling intermediates that were either significantly activated, or ignored, upon BCR stimulation of cells. Therefore, starting from the BCR, we next traced all the possible shortest paths leading to each human ortholog of the signaling intermediate that was shown to be activated in Figure 1B. Here, we considered a signaling intermediate to be activated only if its phosphorylation levels were increased by at least 2-fold in response to anti-IgM stimulation. This filtration exercise short-listed Raf1, ERK-1/2, MEK-1/2, p38, JNK, CAMKII, Lyn and Akt1 as the target nodes, and all the resulting shortest paths originating from the BCR to each of these intermediates were merged to create a sub-network.

In order to complete the above network we again employed the shortest path algorithm to next trace the various possible shortest paths from each of the activated signaling intermediates to the set of seven short-listed TFs described in Figure 3B. These paths were then merged to yield the shortest path network from the signaling intermediates to the TFs.

In the final step we merged the three sub-networks comprising of the links between the BCR and the signaling intermediates, the signaling intermediates and the TFs, and the DOR between the TFs and the target genes described in Figure 3B. This synthesis generated an information-centric network that captured the pathways mediating BCR-dependent cell cycle arrest of CH1 cells. The resulting network was comprised of 163 nodes and 416 edges and is depicted in Figure 3. Here, 44 of the constituent nodes (highlighted in grey) are transcription factors whereas 103 (highlighted in blue) are signaling molecules. It is pertinent to note here that the network shown in Figure 3C is distinct from the more conventional protein-protein interaction (PPI), or, gene-regulatory networks in that it represents a hybrid of both approaches. Thus while the links from the BCR through the signaling intermediates and to the TFs essentially constitute a PPI network, the downstream component incorporating links from TFs to the target genes - however - describes a set of protein-to-gene interactions.

### Extracting the gene expression signature of the cellular response

Our next goal then was to delineate the core pathways or modules in the network described in Figure 3C, that specifically regulated the cellular phenotypic response. To do this, however, it was first necessary to identify those BCR-dependent genes described in Figure 3B, that were responsible for enforcing G1 arrest of cycling CH1 cells. Here, we took advantage of our earlier studies in which we had determined the early BCR-dependent gene expression profile in A20 cells. While these latter cells also represent a murine B-lymphoma line these, however, are derived from mature B-cells and are characterized by the memory phenotype [1]. Further, BCR-stimulation of these cells (with anti-IgG) had no effect either on their survival, or on their ability to complete the individual stages of the cell cycle [41].

**Figure 4:**
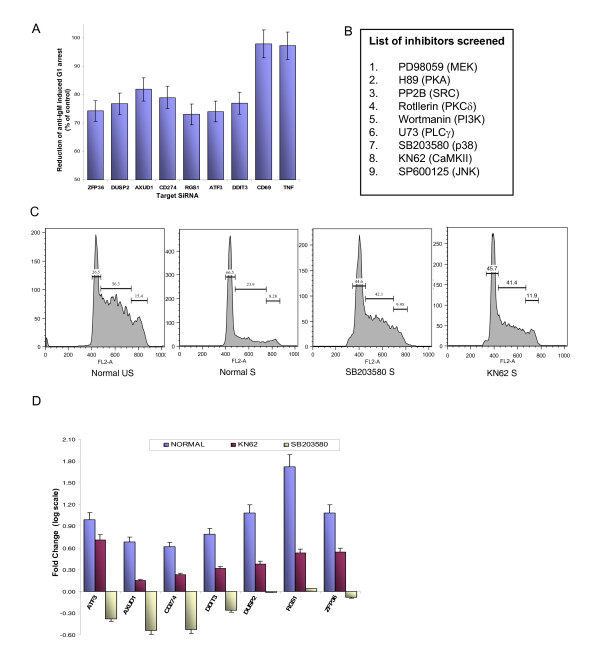
**Identifying the key molecular intermediates involved in mediating BCR-dependent cell cycle arrest**. Panel A shows the population of G1 arrested cells under RNAi mediated knockdown of early induced genes identified as immature B cell specific. The plot represents mean of three individual replicates with ± S.D. Panel B lists chemical inhibitors screened for protection of CH1 cells from anti-IgM mediated G1 arrest to identify potential regulatory signaling intermediates. Panel C shows the histograms of live population of cells in each of the cell cycle phases under normal anti-IgM stimulation, in presence of p38 inhibitor SB203580 and CaMKII inhibitor KN62. The decrease in the population of G1 arrested cells in both the cases of SB203580 and KN62 could be clearly observed in the histograms. Effect of SB203580 and KN62 on early induced genes one hour after anti-IgM stimulation is shown in panel D. The plot indicates mean value of the fold change in the mRNA levels of the respective genes in log scale obtained from three replicates with ± S.D.

Interestingly, four of the upregulated genes (*EGR1, FOSB, CD69, TNFa) *described in Figure 3B were also found to be induced in A20 cells that had been stimulated through the BCR for 1 h [1] (Accession No. E-TABM-360). This suggested to us that the products of these four genes were unlikely to contribute towards the G1 arrest of CH1 cells. As a result, the list of possible mediators of the BCR-dependent CH1 cellular response could be reduced to the seven genes that remained (i.e. *AXUD1, DDIT3, CD274, ATF3, DUSP2, RGS1, ZFP36)*. To evaluate the possible relevance of members of this latter group, we resorted to a functional approach that involved siRNA-mediated silencing. That is we employed target-specific siRNA to silence the BCR-dependent expression of each of these seven genes, and examined for the consequences on the cell cycle arrest response (Additional File 1: Supplemental Fig. S2). Figure 4A shows that silencing of each of these genes resulted in a partial inhibition of the anti-IgM-induced arrest of cycling CH1 cells. As opposed to this, silencing expression of either *CD69 *or *TNFα *- BCR-dependent genes that are induced in both A20 and CH1 cells - had no such effect (Figure 4A). These results, therefore, support that the effects of BCR stimulation on cycling CH1 cells is, at least in part, mediated through the collective effects - either additive or cooperative - of the seven target genes short-listed above (see Figure 4A). Thus, in other words, induced expression of these target genes likely represents the signature of the anti-IgM-mediated response of cell cycle arrest in CH1 cells.

It is pertinent to note here that our inferred role for these seven target genes as contributors towards cell cycle arrest is also consistent with their known functions in the literature. Thus, *ZFP36 *has recently been described to mediate as an inhibitor of G1 to S progression in pro-B cells [42], whereas either anti-proliferative or pro-apoptotic roles have been described for both *DUSP2 *and *AXUD1 *[43,44]. The product of the *RGS1 *gene has been shown to function as a negative regulator of G-protein coupled receptor signaling and, therefore, has been implicated in inducing apoptosis [45]. In a similar vein, *ATF3 *is a known repressor of transcription and is involved in regulation of apoptosis in several cellular systems [46], while *DDIT3 *causes G1 arrest under cellular stress conditions through binding with CDK2 [47]. CD274 is also alternatively known as programmed cell death ligand-1 (PD-L1) and its receptor PD-1 functions as an immunoinhibitory receptor that is primarily expressed on B-lymphocytes, T-lymphocytes and myeloid cells [48]. The effects of PD-1 engagement by PD-L1 have primarily been studied in T cells where inhibition of proliferation has been observed [48]. Interestingly, an analysis of the gene expression profile in unstimulated CH1 cells revealed that PD-1 was also constitutively expressed in these cells (Accession No. E-TABM-360). This raises the likelihood that the anti-IgM-induced expression of PD-L1 may initiate intercellular interactions where PD-L1 engages and, therefore, activates the constitutively expressed PD-1 on the neighboring cell.

### Resolving the response-specific BCR-dependent cell regulatory network

Having defined the gene expression signature for inducing cell cycle arrest in stimulated CH1 cells, we next wanted to describe the sub-network of signaling pathways that mediated the regulation of these genes. To do this we adopted an approach in which perturbations were introduced in the BCR-dependent signaling network through the selective inhibition of several of the constituent nodes. The consequences of this inhibition on the anti-IgM induced cellular response were then monitored. Node-specific inhibition was achieved by the use of pharmacological inhibitors and these, along with their kinase-specificity are listed in Figure 4B. Experiments in which CH1 cells were stimulated with anti-IgM in the presence of each of these inhibitors revealed that only KN62 and SB203580 - highly specific inhibitors of CAMKII and p38 MAPK respectively [49] - were able to reverse the block in cell cycle progression to any significant extent (Figure 4C). In contrast addition either of wortmannin, rottlerin, or U73 led to an increase in cell death even in the absence of any stimulation, whereas none of the remaining inhibitors had any effect on the anti-IgM-dependent G1 arrest (data not shown).

Since KN62 and SB203580 were both able to inhibit the effects of anti-IgM, we also examined for their effects on IgM-dependent gene expression. CH1 cells were stimulated either in the presence or absence of these inhibitors and the consequent expression of the seven cell cycle-regulatory genes short-listed from Figure 3B was determined by quantitative RT-PCR (Additional File 3). The results obtained are summarized in Figure 4D. As shown, both pharmacological agents inhibited BCR-dependent induction of all seven genes although the effects of SB203580 were significantly more potent than that of KN62 (Figure 4D). The inhibitory effect of KN62 ranged from modest (*ATF3*) to significant, whereas that of SB203580 ranged from one that was near quantitative (*DUSP2, RGS1, ZFP36) *to a marked repression to below basal levels (*ATF3, AXUD1, CD274, DDIT3*) (Figure 4D). Thus the ability of the CaMKII inhibitor KN62, and that of the p38 inhibitor SB203580 to prevent BCR-dependent cell cycle arrest correlates with their ability to also block expression of the genes that presumably drive this response. Interestingly, although SB203580 was more potent than KN62 at inhibiting anti-IgM-specific gene expression (Figure 4D), both compounds were - nonetheless - similarly effective at inhibiting the cell cycle arrest response (Figure 4D). This may suggest a relatively high threshold of vulnerability for the products of these genes, with the magnitude of the reduction in their levels achieved by KN62 being sufficient to neutralize their effects on the cell cycle. Consequently then, the greater potency of SB203850 - at the level of gene expression - would not necessarily translate into a greater inhibition of the effects of anti-IgM on the cell cycle.

### Dissection of the perturbations induced by KN62 and SB203580 on the BCR-signaling network

**Figure 5:**
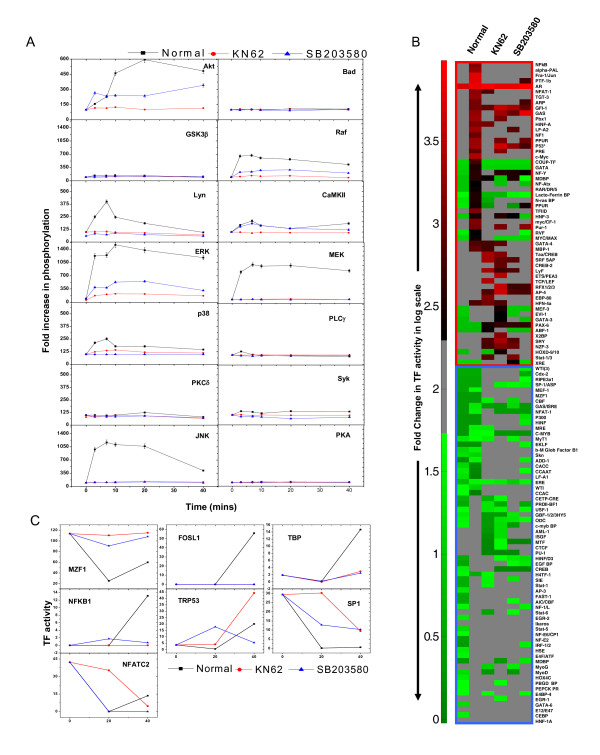
**Delineating the core molecular constituents of the BCR-dependent pathways mediating cell cycle arrest**. Panel A depicts the time dependent phosphorylation status of signaling intermediates upon anti-IgM stimulation in presence of p38 inhibitor SB203580 and CaMKII inhibitor KN62. Values are mean of three individual experiments with ± S.D. Modulation of Transcription factor activities upon BCR stimulation in the presence of SB203580 and KN62 is shown in panel B. The TF array blots after quantification and normalization are represented as heat map to represents the fold changes in TF activity expressed on a log_10 _scale. Panel C shows the activity profiles of seven TFs short listed based on their effect of early-induced genes in presence of the p38 and CaMKII inhibitors (see text for details). Shown here are the actual quantitative numbers obtained after image quantification.

Since we had two separate inhibitors that similarly suppressed the effects of anti-IgM stimulation, it became possible to use these to dissect the core pathways involved. CH1 cells were stimulated with anti-IgM either in the presence or absence of either SB203580 or KN62, and the effects on time-dependent phosphorylation of the fourteen BCR-sensitive signaling intermediates (see Figure 1B) was monitored (Additional File 1: Supplemental Fig. S1). The resulting normalized and quantified profiles obtained are shown in Figure 5A.

Interestingly, although highly specific for p38 [50], the addition of SB203580 led to a significant inhibition in BCR-dependent phosphorylation of all the signaling intermediates examined (Figure 5A). The only exception here was CaMKII where the inhibitory effect was marginal (Figure 5A). A similar effect of a near global inhibition of BCR-dependent signaling was also observed in response KN62 addition (Figure 5A). In this case, however, phosphorylation of p38 was also inhibited (Figure 5A). That is, while inhibition of CaMKII also led to attenuation of p38 phosphorylation the reverse was, however, not the case (Figure 5A).

While a mechanistic explanation for these differential effects was not immediately obvious these latter results, nonetheless, suggested that the effects of CaMKII inhibition may also be mediated through the consequent inhibition of p38 activation. Here it must be emphasized that both KN62 and SB203580 are reported as highly specific inhibitors of CaMKII and p38 activity thus excluding the possibility of off-target effects that might arise [49].

The perturbations in the BCR-signaling network induced by these two pharmacological inhibitors also resulted in profound effects at the level of the BCR-sensitive TFs. This is apparent from the heat map shown in Figure 5B, which compares the fold change in activity of individual TFs at 20 and 40 minutes of stimulation with anti-IgM either in the absence or presence of these inhibitors. The green and red maps represent a >2-fold repression and activation respectively, while the grey map is indicative of no change in activity. Broadly speaking, it is evident that both the inhibitors employed significantly attenuate the effects of anti-IgM, during either the enhancement or suppression of TF activities (Additional File 1: Supplemental Fig. S3).

For our further analysis, we concentrated on examining the activation profiles of only those seven TFs that were short-listed in Figure 3B. This was because our primary aim was to extract the pathways through which signal perturbation by KN62 and SB203580 influenced the gene expression pattern observed in Figure 4D. As shown in Figure 5C, stimulation of cells with anti-IgM led to repression in the activity of MZF1. This inactivation, however, was inhibited in the presence of both KN62 and SB203580 (Figure 5C). Similarly, the anti-IgM induced inactivation of Sp1 was also partly inhibited by SB203580, whereas this inactivation was delayed in the presence of KN62 (Figure 5C). Somewhat surprisingly, stimulation also resulted in a rapid reduction of the basal activity of NFATc2. Further, while inhibition of p38 had no significant effect on this process, the inclusion of KN62 lead to at least a delay in the kinetics of this inactivation (Figure 5C). In contrast to these repressive effects, BCR-crosslinking also induced a delayed enhancement in the activities of FOSL1, TBP, NFKB1 and - to lesser extent - TRP53 (Figure 5C). However, inhibition of either CaMKII or p38 led to a near complete suppression of this activation in the case of FOSL1, TBP, NFKB1, but not of that of TRP53 (Figure 5C). Thus, in at least four of the seven cases, both inhibitors exerted comparable effects on their anti-IgM-induced activation profiles. The reasons for the differences observed in the remaining three TFs are unclear at the present time. Notwithstanding this however, the results in Figure 5C permitted us to infer that the four similarly affected TFs - MZF1, FOSL1, TBP, and NFKB1 - could at least partly rationalize the overlapping effects of these two inhibitors on anti-IgM-stimulated cells, both at the level of gene expression and cell cycle arrest.

### The p38 MAP kinase influences BCR signaling through a constitutively active feedback regulation of Lyn

The results in Figure 5A that inhibition of p38 led to a concomitant inhibition of nearly all the BCR-dependent signaling intermediates was particularly intriguing. Importantly, this also included the protein tyrosine kinase Lyn. The *src *kinase family member Lyn represents one of the earliest kinases recruited by the BCR, the activation of which then ensures activation of the downstream signaling pathways [51-54]. Consequently, suppression of Lyn activation by p38 inhibition offered a simple explanation for the near global effect of SB203580 on BCR-signaling. In other words, these results suggested the likely existence of a positive feedback regulatory loop where p38 also influences Lyn activation. Relevant to this was the finding in Figure 5A that addition of SB203580 induced a reduction in phospho-Lyn levels even in the absence of any stimulation of cells with anti-IgM. That is, p38 may constitutively interact with Lyn even in the absence of BCR engagement.

**Figure 6:**
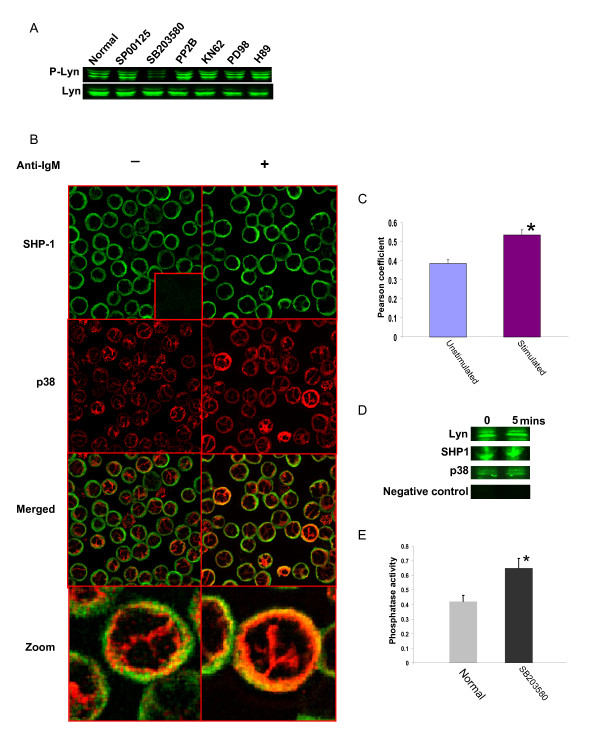
**The MAP kinase p38 indirectly regulates the activation status of Lyn**. Shown in panel A are phosphorylation levels of Lyn in presence of a panel of inhibitors with Lyn molecule as loading control. The images in panel B confirm the existence of a co-localized pool of p38 and SHP-1 in unstimulated CH1 cells. Further, increase in the extent of co-localization was also observed after anti-IgM stimulation for 5 min. Panel C shows the Pearson's co-localization coefficient for p38 and SHP1 obtained from the images in panel B. Values obtained in both unstimulated and anti-IgM-stimulated (5 min) are plotted. Experiments were performed in triplicates and mean value ±S.D. is plotted (* indicates p-value < 0.05). Panel D shows the results of an experiment where the BCR was immunoprecipitated from either unstimulated, or anti-IgM-stimulated (5 min) cells. Immunoprecipitates were then resolved by gel electrophoresis and then probed for the presence of either p38, SHP-1 or Lyn by Western blot analysis. Results shown confirm that all of these three molecules were associated with the BCR in both unstimulated and stimulated cells. In addition, these results further confirmed the findings of p38-SHP-1 co-localization in panel B and C. The negative control shown represents a Western blot for Lyn in samples where mouse IgM conjugated to agarose was used for the immunoprecipitation. A similar negative result was also obtained when these samples were probed for presence of either p38, or SHP-1 (not shown). Panel E shows phosphatase activity present in immunoprecipitates of the BCR that were obtained from unstimulated cells either in presence or absence (Normal) of the p38 inhibitor SB203580 (Materials and Methods). The assay was performed in triplicates and the mean value (± S.D.) is given, * indicate statistically significant (p-value < 0.05) increase in phosphatase activity in presence of SB203580.

To verify this we first tested the effects of a panel of pharmacological inhibitors, including SB203850 and KN62, on the basal phosphorylation of Lyn in CH1 cells. As shown in Figure 6A, none of these inhibitors had any significant effect on intracellular concentrations of the Lyn protein. Levels of its phosphorylated form were, however, markedly reduced in cells treated with SB203580. Importantly, this effect on Lyn phosphorylation was specific for SB203580 with none of the other inhibitors tested, including KN62, showing such an activity (Figure 6A). Thus, at least in CH1 cells, the Ser/Thr kinase p38 appears to regulate the basal activation status of the protein tyrosine kinase Lyn.

We have previously shown that the phosphorylation state of Lyn in B cells is governed by the activity of the BCR-associated protein tyrosine phosphatase (PTP) SHP-1 [51]. Interestingly, studies from other groups have demonstrated that the activity of SHP-1 can, in turn, be regulated through phosphorylation at specific Ser/Thr residues [55]. It, therefore, seemed plausible to us that p38-dependent modulation of basal phospho-Lyn levels may represent an indirect effect that is mediated through SHP-1. That is, the enhanced basal levels of activated Lyn could represent a consequence of attenuated SHP-1 activity, which is enforced through its phosphorylation by p38. The possibility of a direct interaction between p38 and SHP-1 was supported by our initial results involving confocal microscopy, which revealed that at least a fraction of the molecules representing these two proteins were indeed co-localized in the proximity of the cell membrane (Figure 6B, C). Subsequent immunoprecipitation experiments established that this co-localized pool also included that subset of SHP-1 that was constitutively associated with the BCR. Thus, a Western blot analysis of BCR immunoprecipitated from either unstimulated or anti-IgM-stimulated CH1 cells also revealed the co-precipitation of both SHP-1 and p38 (Figure 6D). Finally, we could further demonstrate that treatment of the BCR immunoprecipitate with the p38 inhibitor SB203580 resulted in a significant increase in the associated phosphatase activity (Figure 6E). These collective results, therefore, confirm that activity of the BCR-associated SHP-1 was indeed under negative control of the co-associated p38. This, in turn, provides a likely explanation for the increased levels of activated Lyn detected in un-stimulated CH1 cells.

### Extracting the core cellular network that mediates BCR-dependent cell cycle arrest CH1 cells

Our results so far had helped to characterize at least some of the intermediates that were involved during anti-IgM induced signal transduction. In subsequent experiments, we were also able to define the key set of TFs that were responsible for translating the pattern of signaling events generated into the expression of those target genes that were, at least primarily, involved in driving the G1 phase arrest. Having thus generated the molecular map of the network emanating from the BCR and extending up to the enforcement of the specific cellular response, we then also identified a feedback interaction between p38 and SHP-1 that functioned, through the regulation of Lyn activity, as a key regulatory motif of this network.

**Figure 7:**
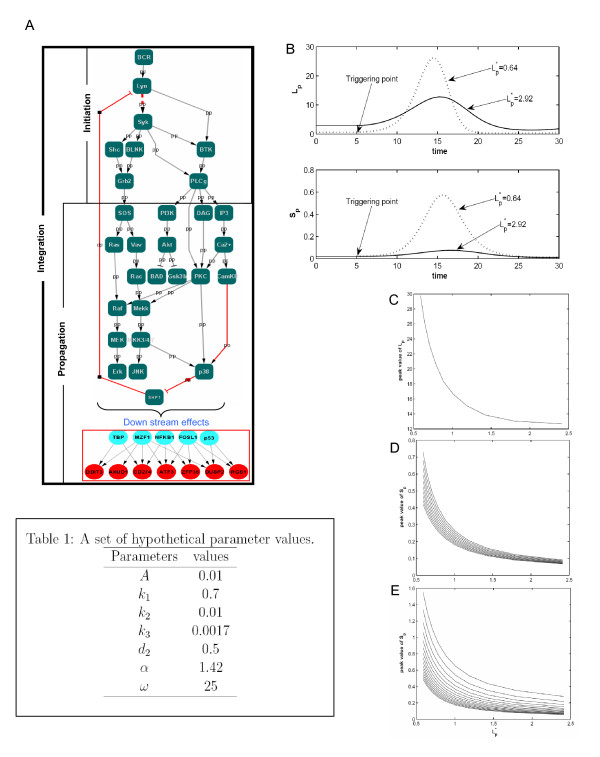
**Defining the core regulatory axis and modeling the role of system homeostasis**. The molecular interaction map that we derived by combining our experimental data with known literature on BCR signaling is shown in panel A (see text for details). The novel feedback regulation of SHP-1 mediated by p38 that we identified is depicted by red edges. The turquoise nodes represent the seven TFs identified by TF array, and the red nodes are the early induced genes specific to CH1 cells. The signaling intermediates are denoted in green nodes and edge label denote either phosphorylation (pp) or dephosphorylation (dp) regulation. The cascade is classified into signal initiation and propagation sub groups for better understanding. Panel B shows different concentrations of the phopho-Lyn and Syk for different basal values of *L_p _*i., e., L_p_*. For the straight line *d*_1 _= 0.25 and for the dotted line *d*_1 _= 1.1, with other parameter values given in the Table 1. Panel C shows the relation between L_p_* and the peak value of the concentration of *L_p _*after stimulation is shown in panel C. Here we vary *d*_1 _from 0.23 to 1.2 in the reverse order, with other parameter values as in the Table 1. Panel D shows relation between L_p_* and the peak value of the concentration of *S_p _*(after stimulation) by varying *d*_1 _for different values of *k*_3 _(from 0.001 to 0.01), with other parameter values as in the Table 1. Panel E shows relation between L_p_* and the peak value of the concentration of *S_p _*(after stimulation) by varying *d*_1 _for different values of *d*_2 _(from 0.1 to 0.8), with other parameter values as in the Table 1.

These cumulative results, therefore, allowed us to further refine the rather generic network map derived in Figure 3C, and obtain a more precise description of the BCR-dependent regulatory network for G1 arrest in CH1 cells. By combining known experimental pathway information on B cell signaling, and the network derived through a shortest path analysis, we could generate the CH1 cell-specific signaling axis responsible for driving G1 arrest (Figure 7A). Further, we could also distinguish the individual stages based on the sequential steps of initiation, propagation, and integration of intracellular signal transduction cascades (see Figure 7A). The step of signal initiation is considered to represent the early events that occur upon receptor engagement, and this early upstream process regulates the downstream targets both qualitatively and quantitatively.

### A mathematical model helps to define key regulators of system homeostasis and Lyn activation

Although we could successfully prove the role of p38 in regulation of Lyn by acting on SHP-1, it did not address the issue of cell type specificity in determining cell fate decisions. It has been reported that levels of phospho-Lyn and other intermediates are significantly higher in immature B cell compared to the mature counterpart cells [52]. We also observed this feature in our present study, when compared with our earlier studies involving mature B cells [1,51]. Since we had sufficient information on both mature and immature counterparts regarding their basal status and the way they respond to the antigen we sought to build a mathematical model representing the fundamental differences in both of these cell types, in terms of the initiation of BCR signaling.

For this we built a model based on a system of ordinary differential equations to analyze Lyn activation and it's down stream effect on Syk activation. Here, while the extent of BCR-dependent Lyn activation by phosphorylation was treated as a measure of the strength of initial signal generated, the magnitude of Syk activation would then define the efficiency of its transduction to the downstream intermediates [51]. The aim here was to identify the key parameter(s) that might lead to weak activation of BCR-dependent signals in immature B cells, as opposed to that in mature B cells. More specifically, we wanted to determine whether the higher levels of basally active Lyn in immature B cells could account for this difference. In our model, we also incorporated the role of SHP-1 as a negative regulator of BCR signaling. It is now widely accepted that receptor-associated phosphatases function as key negative regulators that keep the system in steady state in the absence of an activating ligand. Following engagement of the receptor, however, there is a transient decrease in the negative regulatory activity of this phosphatase, after which it again returns to its initial value [51]. Thus, if *L_p _*denotes the concentration of activated Lyn molecule; *S_m _*that of the Syk molecule susceptible to activation by phosphorylation; and *S_p _*denotes the concentration of activated Syk, the model - with the meaning of each parameter - can be written as follows:

(1) $$\begin{array}{l} \frac{dLp}{dt}=k1+k2Lp-d1g(t)Lp,\\ \frac{dSm}{dt}=A-k3SmLp,\\ \frac{dSp}{dt}=k3SmLp-d2Sp \end{array}$$

With initial conditions *L_p _*(0) ≥ 0, *S_m_*(0) ≥ 0, *S_p_*(0) ≥ 0. *A *is the total amount of Syk molecule present in the cell susceptible to activation; *k_1 _*is the membrane associated Lyn at basal level; *k_2 _*represents the rate at which activation of Lyn take place after the binding of agonist to the membrane receptor; *k_3 _*is the rate at which Syk is activated by the Lyn; *d_1 _*and *d_2 _*represents the amount of negative regulator on the active forms of Lyn and Syk respectively at their ground state.

To take into account the particular nature of the negative regulator as discussed above we assumed,

$$g(t)=1-\alpha \sin\frac{2\pi t}{\omega }$$

Here *α *is the forcing term representing the strength of the stimulation. At ground state *α *= 0.

In unstimulated cells, there is an equilibrium maintained between the active and inactive state of the signaling molecules such as Lyn and Syk. However, it must be emphasized here that there are indeed multiple states of signaling intermediates (like Lyn, Syk) present in the real system due to multiple phosphorylation sites. But our focus here was to analyze the balance between the active and inactive forms of the species and their differential response to varying basal activity. Therefore in the considered scenario, mathematically, at ground state i.e., *α *= *0*, we define the basal value of active-Lyn (*L_p_*) denoted by L_p_* as the value where,

$$\frac{dLp}{dt}=0$$

Using the definition of L_p_* if we solve the equation (1) we get the value of L_p_* as given below (detailed calculations provided in (Additional file 1),

$$Lp*=\frac{k1}{d1-k2}$$

Thus our mathematical result suggests that, at ground state, the basal value is inversely related to the magnitude of the negative regulator acting on the activated Lyn molecule.

We next simulated our model system (1) (the parameter values and the method of simulation are described in Additional file 1) and the results for Lyn and Syk activation are shown in Figure 7A and 7B. Here, we calculated the value of L_p_* from the derived relation. We found that the influence of the negative regulator on Lyn and Syk activity produced two different basal states for them before the trigger point, which denotes BCR-dependent stimulation. But the interesting prediction from the model was that the sensitivity of either Lyn or Syk response to BCR-engagement was significantly higher when the basal activity of Lyn was lower. This finding would be consistent with the experimental results obtained in the present study. To further delineate the effect of basal activity on receptor-induced signaling, we plotted different peak values with respect to different basal values for Lyn and Syk by varying the parameter for negative regulator. Figure 7C shows the result of the simulation where an inverse relation between basal level of Lyn activity, and the extent of its activation after BCR stimulation is clearly evident. To observe the effect of other parameters especially those acting on the active Syk species we performed a similar analysis on Syk. A similar qualitative behaviour with different slope values was again obtained (Figure 7C, D). The results of our modeling analysis thus further substantiated our experimental results by highlighting the role played by the negative regulators of signal initiation, such as SHP-1, in determining the cell fate decision.

## Discussion

B-lymphocytes represent a good model system to study plasticity in receptor-activated signaling processes, and the consequent influence on the cellular phenotypic response. Depending on their state of maturation, antigen encounter by the B-lymphocytes can lead to varied outcomes that range from activation and/or proliferation to anergy, or also to activation induced cell death (AICD) through apoptotic mechanisms [56]. In general, mature B-lymphocytes undergo activation followed by proliferation upon induction of BCR-dependent signaling by an antigen [57,58]. In contrast, engagement of the BCR induces AICD - preceded by an arrest of the cell cycle - in immature and transitional stage immature B cells [41,56]. This latter process serves to eliminate self-reactive B cells during its different stages of development [41,56]. Various cell lines such as WEHI-231, CH31, and B104 among others have been employed as models systems for the study of BCR signaling in immature B cells. In all of these cases, cell stimulation with a suitable surrogate antigen leads first to G1 cell cycle arrest, which is then followed by apoptosis [56,59].

Both the results presented here as well as those described in earlier studies confirm that CH1 cells represent yet another good model system for recapitulating BCR-driven responses in immature B cells. First, similar to immature and transition stage immature B cells, CH1 cells also express high levels of the IgM class of the BCR, with little or no expression of those belonging to the IgD class [11,12]. In immature B cells, BCR-activated cells fail to enter into the S phase and this effect can be reversed by treatment with IL4 [60]. As we have previously shown, CH1 cells also exhibit similar properties [20]. BCR activation shows contrasting effects on p27 expression in mature versus immature B cells. Mature B cells express high levels of p27, which is then downregulated by antigenic stimulation [56]. The situation is reversed in the case of immature B cells where, while the basal levels of this protein are low, BCR engagement leads to rapid upregulation [56]. As shown in this study, CH1 cells also accurately recapitulate this latter situation. In transitional immature B cells, antigenic stimulation leads to a transient activation of the downstream signaling components including that of Akt/PKB and those belonging to the MAP kinase pathway [53]. This feature was also evident in our present examination of BCR signaling in CH1 cells. Finally, the greater extent of ERK phosphorylation relative to that of JNK and p38 observed here was yet another property that is characteristic of antigen-stimulated immature B cells [61]. Thus these comparisons collectively confirm the suitability of CH1 cells as a model for studying mechanisms regulating BCR-induced cell cycle arrest and subsequent apoptosis in immature, transitional stage, B-lymphocytes.

An important aspect of our present study was the systems approach that we adopted, which integrated extensive experimentation with graph theoretical analysis and mathematical modeling. It was the synthesis of these diverse methodologies that enabled us to eventually obtain a comprehensive view on both the quantitative and qualitative features of the BCR-dependent signaling network. In addition it also facilitated a description of the consequent changes in the transcription regulatory machinery, and the downstream effects on changes in expression levels of those genes that eventually contributed towards enforcing a G1 phase-specific arrest of the cell cycle. Of particular note here was our finding that the cellular response was, in all likelihood, a direct consequence of the selective and transient activation of the BCR signaling network. Thus, of the twenty molecules examined, we were only able to observe BCR-dependent phosphorylation for fourteen, with no significant effects being evident for the remaining six molecules. This latter group included the adaptor molecules SHC and BLNK, the anti-apoptotic protein Bcl2, the NF-*k*B activating kinase IKKa, and the cellular kinases Pyk2 and PDPK1. While the absence of phosphorylation of Bcl2 and IKKa may not be surprising in view of the pro-apoptotic response induced by anti-IgM, that the adaptor molecules SHC and BLNK were also not phosphorylated was - however - particularly intriguing. At least in mature B cells, both of these scaffolding proteins play a key role in the assembly of BCR-dependent signaling complexes on the cytoplasmic side of the cell membrane, and are important for fine-tuning BCR signaling to direct appropriate cell fates [62,63]. Even in instances where the extent of anti-IgM-induced phosphorylation was more significant, this was only transient in most cases with levels of the respective phospho-protein progressively declining after reaching their peak value. The weak perturbation of the transcription regulatory network, leading to a biased expression of those early response genes that were involved in the cell death pathways, was presumably a direct outcome of the sparse nature of the BCR signaling network in these cells.

We believe that successful extraction of the core BCR-dependent regulatory network that enforced cell cycle arrest in CH1 cells represents a key highlight of our study. Its significance lies in the fact that this network encompasses pathways emanating from the BCR to the key signaling intermediates, and then also those extending from these intermediates to the TFs that were critical for inducing expression of the pro-apoptotic genes. This could be achieved by employing an *in silico *based network approach that combined the data on BCR-activated signaling events, with that on modulation of TF activities. Further, this approach also enabled us to integrate the DOR motif that linked these TFs to the effector genes. Importantly here, the effector genes responsible for causing G1 arrest could first be identified through a comparison of the early gene expression profile between CH1 and mature B cells, and then functionally verified in experiments involving their selective depletion by siRNA.

Having delineated the core BCR-dependent molecular network that specified the G1 arrest, we could then test the effects of specific perturbations so as to identify the key signaling intermediates involved in driving this response. By using a panel of pharmacological inhibitors against different kinases, we localized p38 and CAMKII as the likely targets. Such an inference could be derived from our observations that, of the inhibitors tested, only those specific for either of these kinases were capable of at least partially reversing anti-IgM-induced G1 arrest of the cells. A subsequent examination of the expression profile of the effector gene subset revealed that p38 inhibition was more effective at inhibiting induction of these genes, thus identifying p38 as the central regulator of the anti-IgM induced cell cycle arrest response.

## Conclusions

Interestingly, the mechanism by which p38 exerted such a prominent effect involved a novel feedback loop that controlled signal amplification at a level that was immediately proximal to the BCR. As we showed, p38 directly regulated activity of the BCR-associated phosphatase SHP-1, which in turn influenced the activity of Lyn, the earliest intermediate involved in BCR signaling. Thus, p38 mediated attenuation of SHP-1 activity led to increased basal levels of Lyn phosphorylation, thereby rendering it less sensitive to BCR activation. The selective and transient activation of the signaling network then was direct consequence of the dampening of the initiating signal from the BCR. This aspect could be further elaborated by the simple mathematical model that we developed to analyze the parameters involved in defining the strength of the initial signal generated. Our model revealed a strong influence of the receptor proximal negative regulator (SHP-1 in this case), which generally balances against positive signals to ensure system homeostasis [51]. By using this model we could confirm that as the basal activation of Lyn increased, due to reduced activity of SHP-1, the sensitivity of this kinase to the BCR also diminished. As a result, transmission of signal to the downstream intermediates was also negatively affected at least when measured at the level of Syk activation.

At one level these latter findings served to rationalize the sparse character of the BCR signaling network in CH1 cells and, by extension, immature B-lymphocytes. In addition to this however, we believe that our revelation of the importance of the basal state of the signaling machinery in defining sensitivity, and thereby the cellular response, to the activation of cell surface also has important bearings from a broader point of view. Thus, differences in the basal phosphorylation state of at least the early signaling intermediates could well explain how variations in the response to the same external stimulus are generated from cells that differ either at the level of tissue type, or activation state [64].

## Authors' contributions

MSJ performed the western blots, TF array profiling, cell cycle analysis and validation experiments, SR conceived the project, analyzed and interpreted the results, NJ performed the cell cycle analysis and western blots. SC built the mathematical model, RD performed the confocal studies. MSJ, SR and NJ prepared the manuscript. KVSR conceived and managed the overall project and contributed to manuscript preparation. All the authors read and approved the final manuscript.

## Supplementary Material

Additional file 1 **This contains the complete List of Additional Files including tables, figures and additional methods**. Click here for file

Additional file 2 **This contains the list of TFs probed by the combo array and their corresponding activation pattern**. This file also contains the information regarding the Entrez Gene IDs of the TFs shown in Figure 2. Click here for file

Additional file 3 **This file contains the complete information of the RT-PCR data and analysis**. Click here for file
