# Supplementary material for: Defining the antigen receptor-dependent regulatory network that induces arrest of cycling immature B-lymphocytes
Source: BMC Syst Biol. 2010 Dec 9;4:169. doi: 10.1186/1752-0509-4-169 (PMC3004859; doi:10.1186/1752-0509-4-169)
Supplement: Additional file 1 — This contains the complete List of Additional Files including tables, figures and additional methods. [file 1752-0509-4-169-S1.PDF]

List of Additional Materials

Index	Description
1. Figure S1	Western blots of sixteen signaling intermediates after anti-IgM stimulation in presence and absence of inhibitors
2. Figure S2	FACS histograms for the siRNA effect on cell cycle
3. Figure S3	Transcription factor array blots
4. Figure S4	RT-PCR for siRNA efficacy of genes
5. Table S1	Table explaining the role of signaling intermediates in our study
6. Table S2	Quantification of TF array blots with or without inhibitor from the TF array .
7. Table S3	Ct Value obtained with or without inhibitor for seven upregulated genes from the RT-PCR.
8. Table S4	Curated Literature reference for TFs regulating upregulated genes.
9. Table S5	List of siRNA used from QIAGEN
10. Table S6	List of Antibodies used from Cell signaling
11. Additional Methods	Details of Mathematical modeling and key words for IPA analysis and a detailed description of RT-PCR

Figure S1A

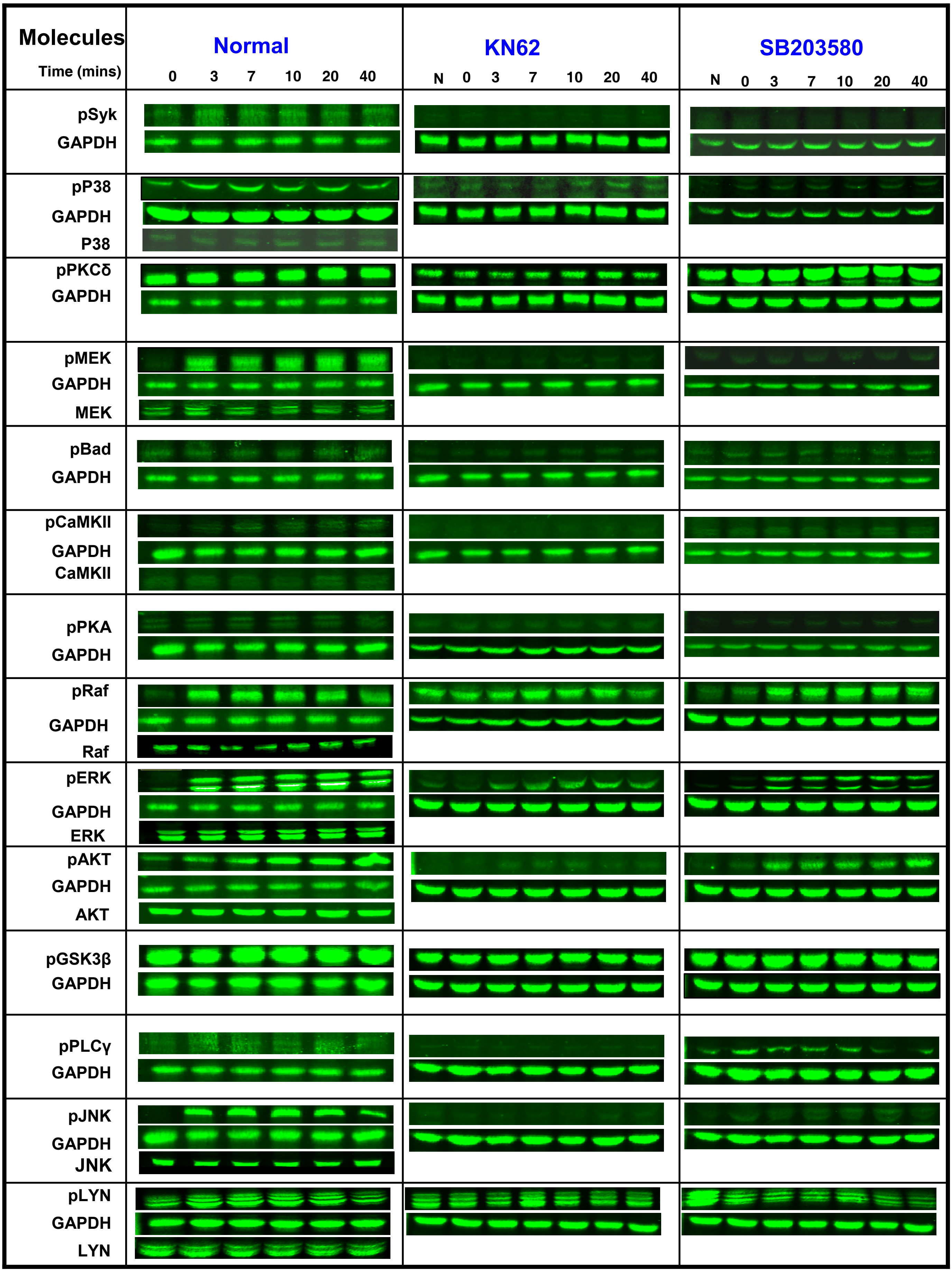
Molecules
Time (mins)
Normal
0 3 7 10 20 40
KN62
N 0 3 7 10 20 40
SB203580
N 0 3 7 10 20 40
pSyk
GAPDH
pP38
GAPDH
P38
pPKCδ
GAPDH
pMEK
GAPDH
MEK
pBad
GAPDH
pCaMKII
GAPDH
CaMKII
pPKA
GAPDH
pRaf
GAPDH
Raf
pERK
GAPDH
ERK
pAKT
GAPDH
AKT
pGSK3β
GAPDH
pPLCγ
GAPDH
pJNK
GAPDH
JNK
pLYN
GAPDH
LYN

Figure S1B

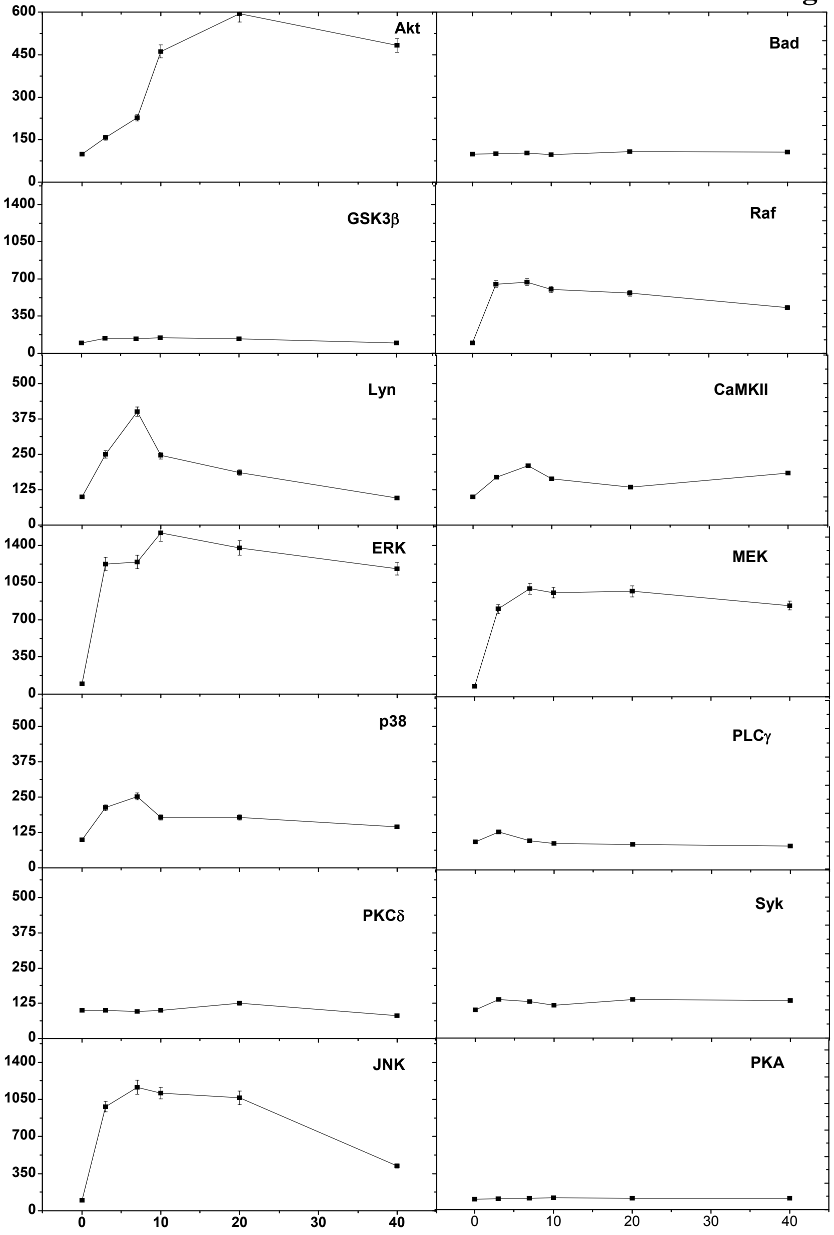

Western profile for the fourteen selected signaling intermediates upon anti-IgM stimulation with or without inhibitors against signaling intermediates.

Figure 1A shows the phosphorylation profiles obtained for the selected signaling molecules from one of three separate experiments. Normal, KN62, SB203580 among three groups represents under anti-IgM stimulation in absence of inhibitor, in presence of KN62 and SB203580 respectively. Cells were treated with inhibitors 20 minutes prior to stimulation.. The Western blots obtained were then normalized as described in main text. Shown are blots for phosphorylated forms of the proteins in comparison with GAPDH controls. In case of anti-IgM stimulation alone i.e. in absence of inhibitors we found eight proteins to be phosphorylated > 2 fold for which we probed for protein levels as a addition control. The normalized protein intensities from blots in the first column were used to plot main Figure 1B. while, blots in second and third column were used to plot main Figure 5C

The plot in Figure 1B corresponds to the quantified phosphorylation profiles of the signaling intermediates after data normalization with the corresponding loading controls. The plot represents mean value of three individual replicates with ±S.D.

Figure S2

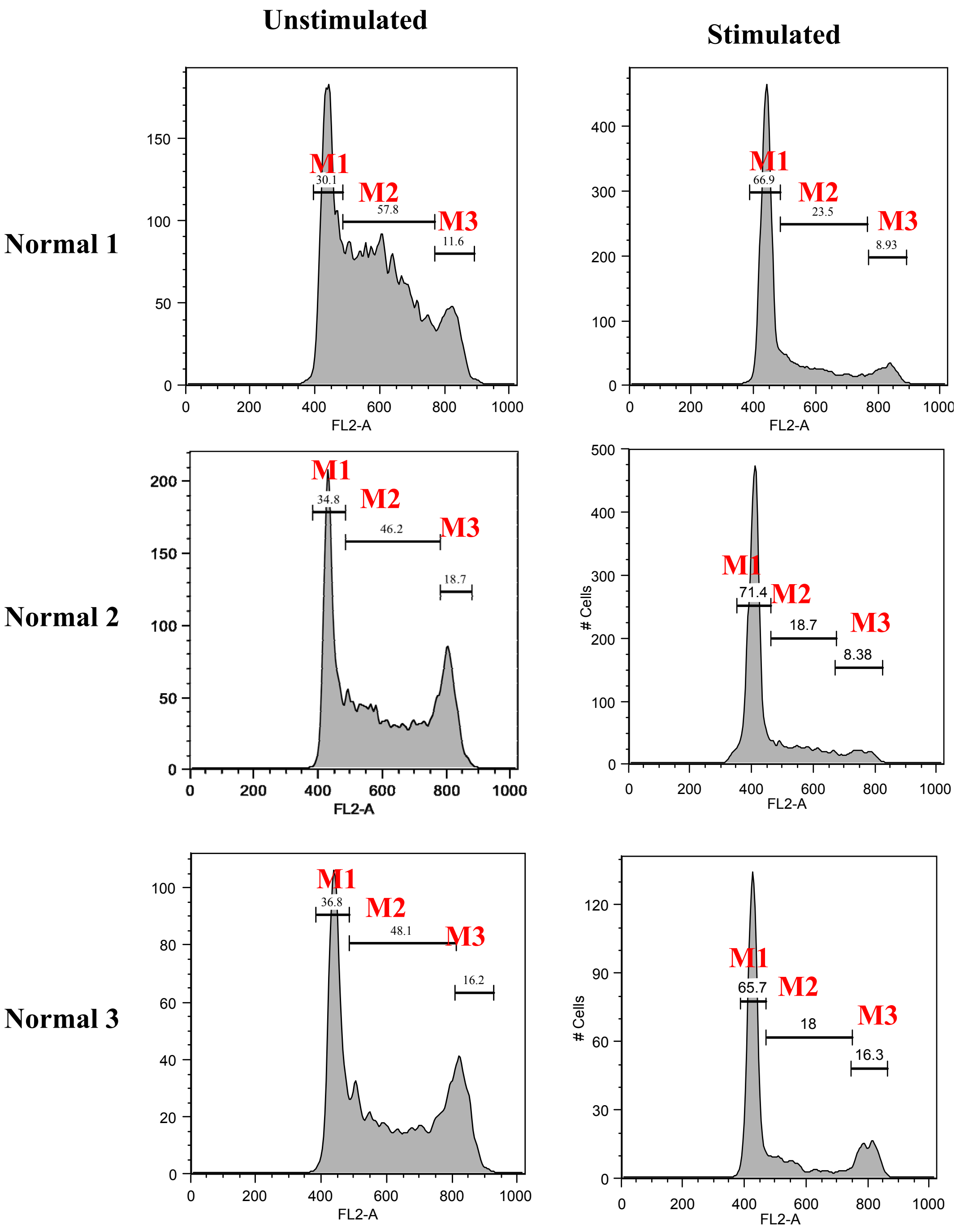

Unstimulated
Stimulated
Normal 1
Normal 2
Normal 3
M1
M2
M3
30.1
57.8
11.6
66.9
23.5
8.93
34.8
46.2
18.7
71.4
18.7
8.38
36.8
48.1
16.2
65.7
18
16.3
FL2-A
Cells

Figure S2
Contd..

Mock Transfected 1

Mock Transfected 2

Mock Transfected 3

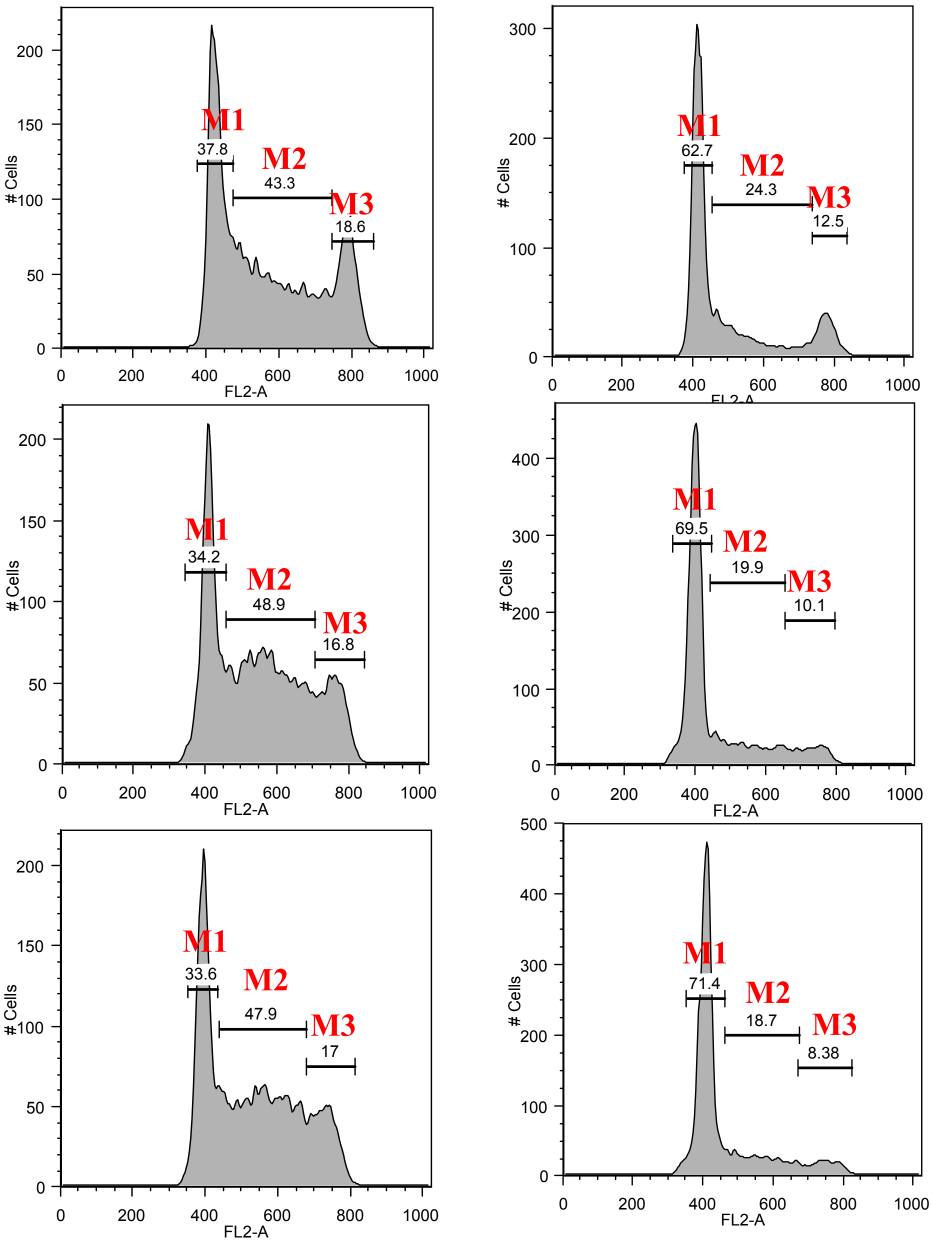

Figure S2
Contd..

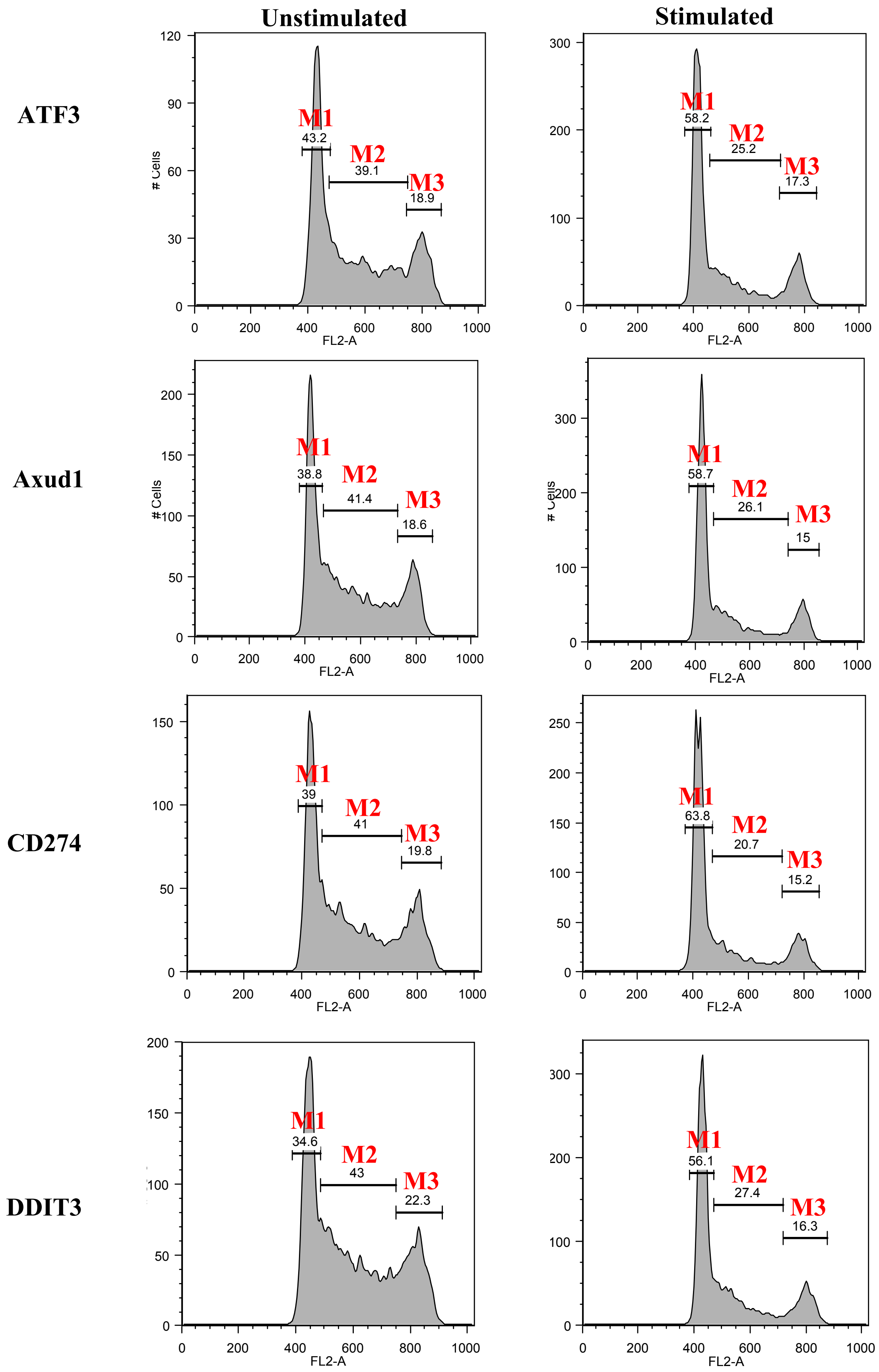

Figure S2
Contd..

Unstimulated | **Stimulated**

Dusp2

RGS1

ZFP36

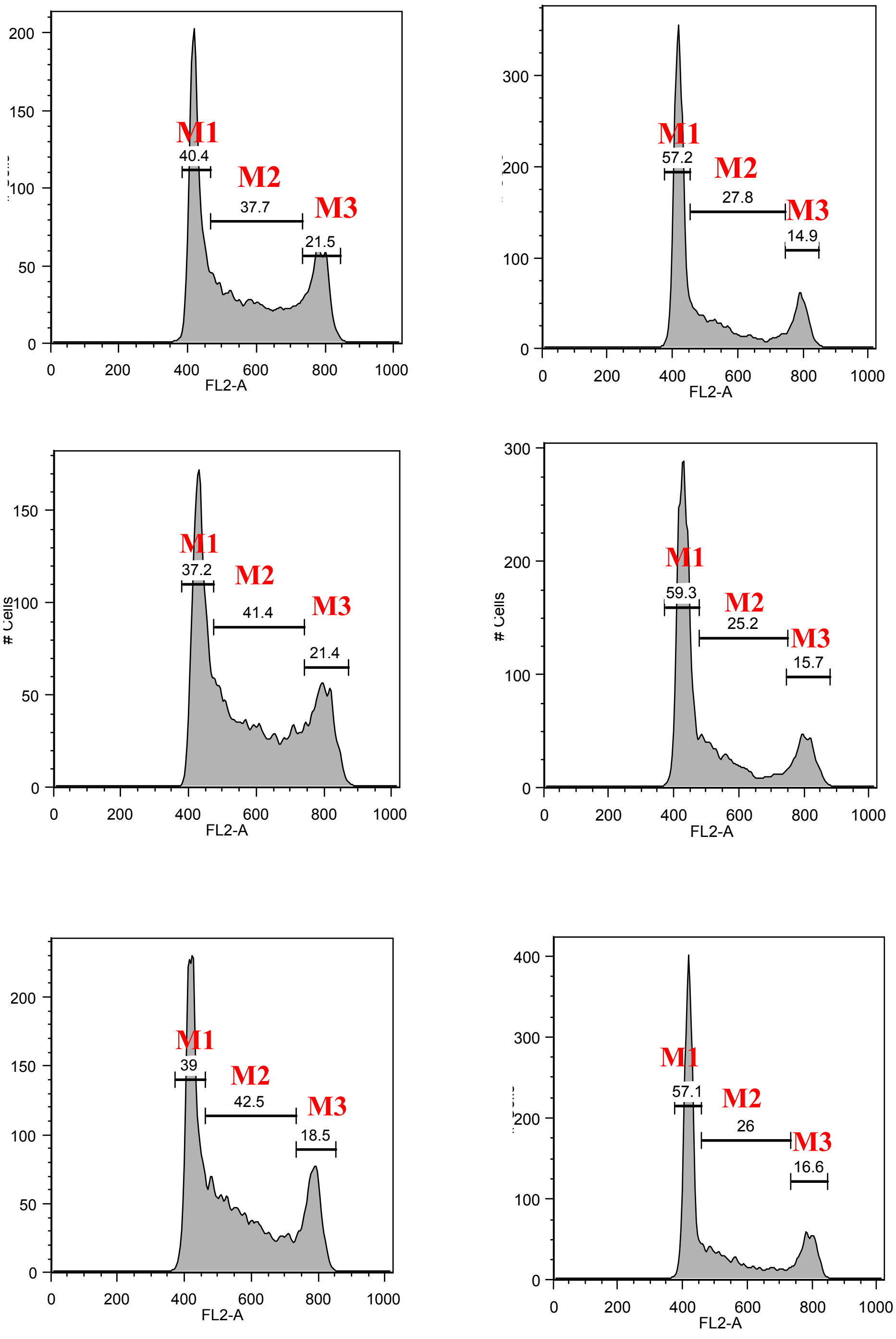

Figure S2
Contd..

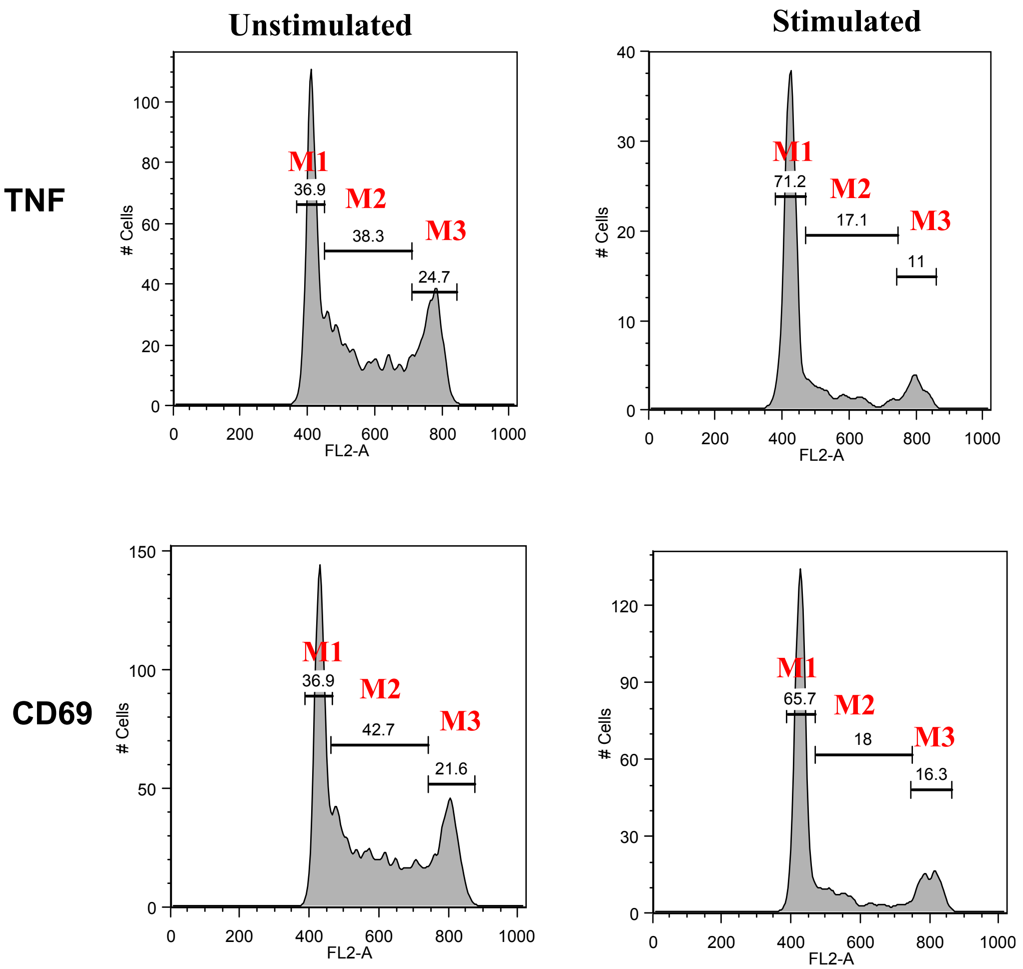

Scanning the role of seven upregulated genes upon anti-IgM induced G1 aarest :
CH1 Cells were treated with siRNA 48 hour prior to stimulation with anti-IgM. After 16 h of stimulation, the cells were harvested and stained with Propidium Iodide to see the role in the regulation of G1 phase by FACS, analysis were performed by FLOJO software. M1, M2 and M3 represent G0/G1, S and G2/M phases respectively and dead or apoptotic cells were not selected for analysis. The extent of cell cycle arrest was determined by measuring the relative proportion of cells in the G0/G1, versus the S and G2/M phases in each of the experimental groups.

Figure S3

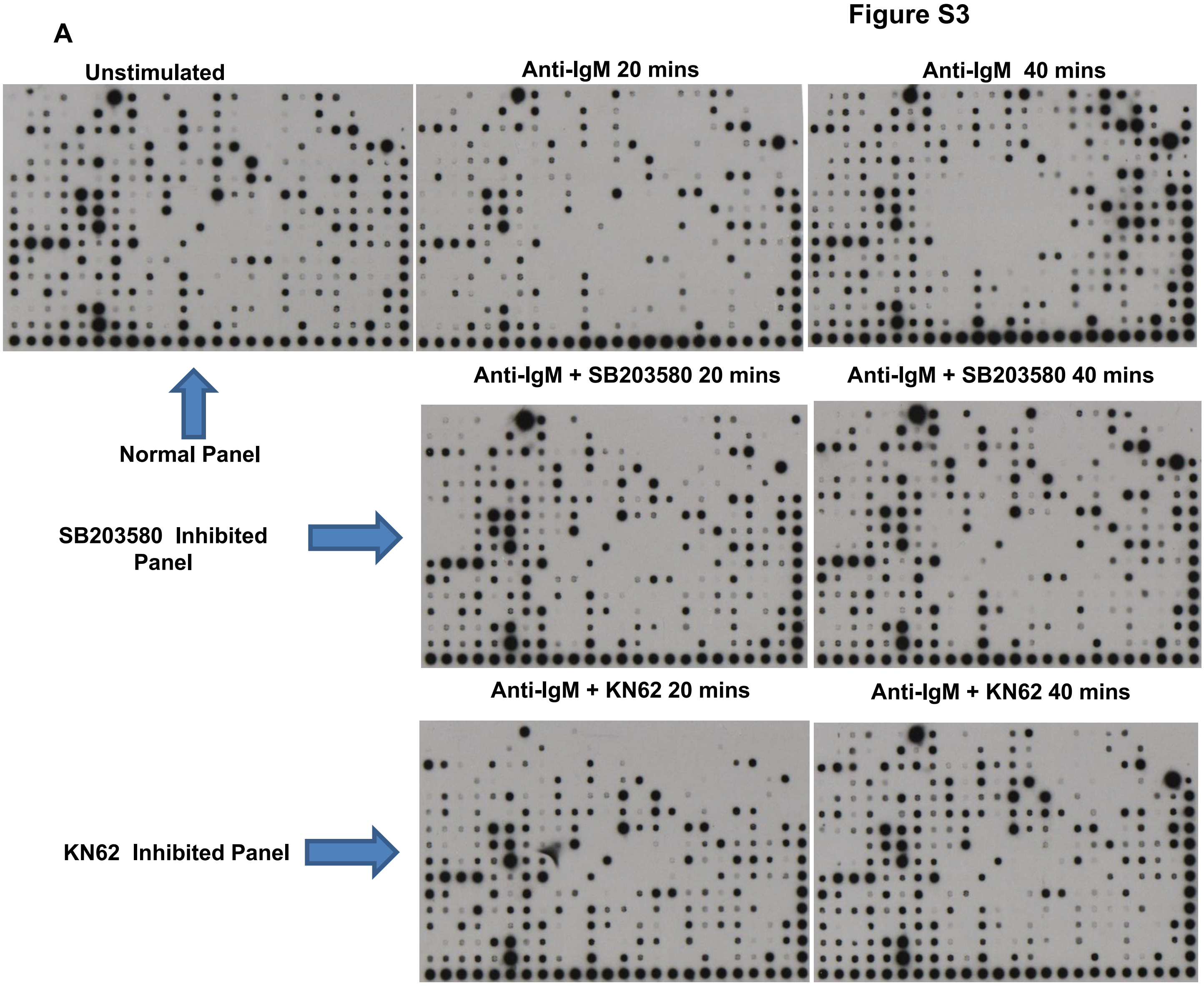

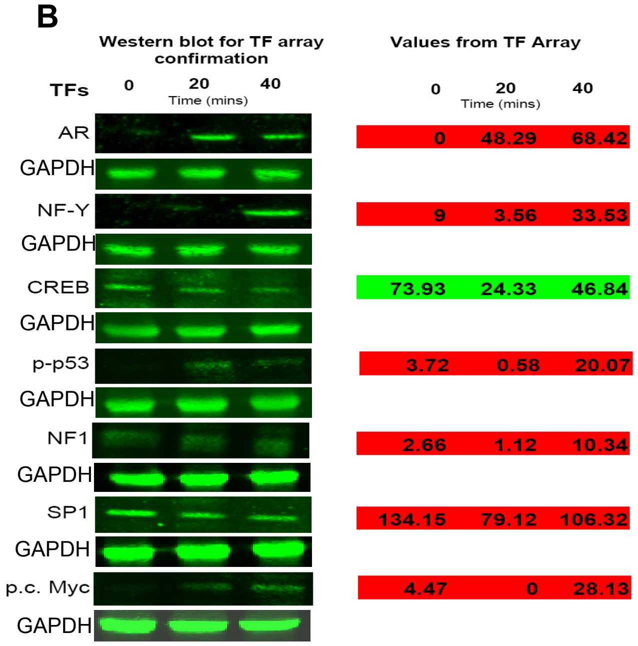

Capturing TFs profile under G1 arrest :
CH1 cells were either left unstimulated (Control), or stimulated under three conditions; in the last two panels SB203580 and KN62, cells were treated 20 min prior to stimulation and the activity profiles of transcription factors determined at 20 and 40 min as described in Experimental methods. Figure shows the representative blots obtained in these experiments. B) CH1 cells were stimulated with anti-IgM for 20 min and 40 min and nuclear extract were made strictly followed by Panomics Nuclear Extraction Kit (Cat No-AY2002) to validate the Combo Array profile for seven of the transcription factors depending on the availability of antibodies in our laboratory just because of cost. Further protocol for western followed as mentioned in material and methods

Figure S4

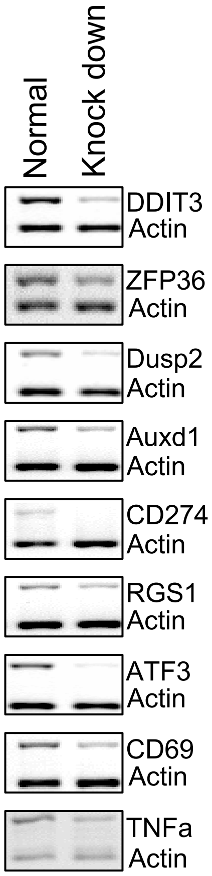

Shown are RT-PCR results validating the RNAi mediated silencing of the targeted genes employed for protection from anti-IgM mediated G1 arrest. The experiment was performed in triplicate yielding similar silencing of the targeted genes and representative blots are shown here. The blots for the respective gene along with their controls (Actin) are represented.

Table S1

List of molecules shortlisted for the study of BCR signaling along with their known function

Common Name	Entrenz Gene Name	Function
CAMK2A	CAMK2A	Serine/threonine kinase, involved in calcium signaling pathway and role in NFAT activation.
PKA	PRKACA	cAMP-dependant protein kinase involved in CREB activation.
PLCg	PLCG2	Phospholipase C, involved in production of IP3 and DAG, thereby controlling calcium release and PKCs activation
AKT	AKT1	Involved in PI3K-AKT pathway. Phosphorylates GSK3b, BAD and provides fine tuning of cellular outputs
ERK1/2	MAPK1	Extracellular signal regulated kinase. Role in phosphorylation of key nuclear targets. A key node in MAPK signaling.
BAD	BAD	Positive regulation of apoptosis by forming complexes with Bcl2 and Bcl-xl.
SYK	SYK	Protein tyrosine kinase, which binds to BCR upon activation to recruit and activate downstream signaling molecules. Key role in signal transduction.
MEK	MAPK3	Key kinase molecule in MAP kinase pathway. Known to regulate activity of intracellular phosphatases by feedback loops for signal regulation.
p38	MAPK14	Critical kinase for cell cycle by regulation of p53, cdc25b and stress related transcriptional events.
C-Raf	RAF1	Downstream molecule of RAS family and involved in phosphorylation of Mek1/2 to activate ERK.
PKCd	PRKCD	Activated by DAG and calcium and involved in diverse cellular signaling events. Regulation of cellular growth, apoptosis in variety of cell types.
JNK	MAPK8	Involved in immediate early gene expression in response to cell stimuli.
GSK3B	GSK3B	Regulation of cell cycle and apoptosis. Involved in export of protein to nucleus.
SHC	SHC	Shc is a adapter protein involved in cell signaling through various types of receptors leading to MAPK activation, c-Myc activation and cell survival.
BCL2	BCL2	B-cell lymphoma 2 plays pro or anti-apoptotic roles depending on activation or inactivation of an inner mitochondrial permeability transition pore, which is involved in the regulation of matrix Ca2+, pH and voltage.
BLNK	BLNK	A cytoplasmic adaptor protein that bridges BCR associated kinase activation with downstream signaling pathways, thereby affecting various biological functions.
PYK2	PTK2B	A cytoplasmic protein tyrosine kinase which is involved in calcium-induced regulation of ion channels and activation of the MAPK.
STAT6	STAT6	Invovled in cell survival activity by inducing BCL2L1/BCL-X(L) under IL-4 stimulation.
IKKa	IKKa	Controls the activation of NF- B transcription factors, which plays pivotal role in various processes like inflammation.
PDK1	PDPK1	One of the major enzymes responsible for the regulation of homeostasis of carbohydrate fuels in mammals by Phosphorylation of (pyruvate dehaydregenase) PDH (that catalyzes the oxidative decarboxylation of pyruvate) which results in inactivation of PDH.

Table- S4

Identification of TFs involved in the particular gene expression by literature survey

Gene Name	TF involved in gene regulation(References)	Description
ATF3	1. c-Myc (S1)<br><br>2. EGR-1 (S2)<br>3. p53 (S3)<br>4. C/EBP (S4)<br>5. NFkB (S5)<br>6. E2F (S5)<br>7. AP-1(S5)	1. ATF-3 gene expression depends on the c-Myc complex at the gene promoter.<br>2. Over-expression of Egr-1 protein induced ATF3 mRNA and Egr-1 siRNA block the induction of ATF3.<br>3. ATF3 protein was induced more efficiently in cells with intact p53 allele than in those with null mutant p53 allele.<br>4. Mutation of the predicted *Atf3 C/EBP site disrupted Atf3 promoter* activation.
DUSP-2	1. P53(S7)<br>2. E2F1(S8)	1. p53 activates the transcription of PAC1 by binding to the PAC1 promoter.<br>2. E2F-1 increases expression of PAC1 at both transcriptional and translational levels
CD274	1. IRF-1(S9)	1. EMSA,site-directed mutagenesis and knockdown experiment revealed that IRF-1 is primarily responsible for the B7-H1.
DDIT3	1. NF-1(S10)<br>2. Maf-A(S11)<br>3. SP-1(6)	1. EMSA and point mutation showed that NF-1 negatively regulates the DDIT3.<br>2. CAT assay and transient transfection shows that AP-1 alongwith ATF-3 is required for DDIT3 regulation.
ZFP36	1. STAT-1(S12)	1. IFN-induced expression of Ttp depends on the IFNactivated transcription factor STAT1.
FOSB	1. CREB(S13)	1. DNA precipitation and DNA decoy experiments indicated that ERK-dependent activation of CREB binding to a CRE/AP-1 like element (designated “CRE2”) at the position of 413 largely contributed to the transcriptional effects of Fos.
EGR1	1. EGR1(S14)<br>2. SP1(S20)	1-2. .EGR-1 is shown to down-regulate the transcription of its own gene expressionw, whereas Spl activated Egr-1 gene expression.
CD69	1. AP-1(S15)<br>2. NFkB(S15)	1-2. CD-69 expression is accompanied by an increased binding of jun and fos family members.
TNF	1. Sp-1(S16)<br>2. NFkB(S17)<br>3. PU-1(S20)<br>4. AP1 & AP2 (S18)<br>5. EGR and NFAT(S19)	1.Sp1 decoy ODNs affect the TNF gene expression.<br>2.NF-KB activation increased the expression of TNF-α-mRNA.<br>3. Real-time PCR, ELISA, and Chip assays confirms the kinetics and magnitude of TNF-α expression levels following LPS- or IgE-stimulation are related to the amount of PU.1 binding to the promoter..

Table S4 References

S1- Tamura, K., B. Hua, S. Adachi, I. Guney, J. Kawauchi, M. Morioka, M. Tamamori-Adachi, Y. Tanaka, Y. Nakabeppu, M. Sunamori, J.M. Sedivy, and S. Kitajima. 2005. Stress response gene ATF3 is a target of c-myc in serum-induced cell proliferation. *Embo J*. 24:2590-601.

S2- Bottone, F.G., Jr., Y. Moon, B. Alston-Mills, and T.E. Eling. 2005. Transcriptional regulation of activating transcription factor 3 involves the early growth response-1 gene. *J Pharmacol Exp Ther*. 315:668-77.

S3- Zhang, C., C. Gao, J. Kawauchi, Y. Hashimoto, N. Tsuchida, and S. Kitajima. 2002. Transcriptional activation of the human stress-inducible transcriptional repressor ATF3 gene promoter by p53. *Biochem Biophys Res Commun*. 297:1302-10.

S4- Huo, J.S., R.C. McEachin, T.X. Cui, N.K. Duggal, T. Hai, D.J. States, and J. Schwartz. 2006. Profiles of growth hormone (GH)-regulated genes reveal time-dependent responses and identify a mechanism for regulation of activating transcription factor 3 by GH. *J Biol Chem*. 281:4132-41.

S5- Liang, G., C.D. Wolfgang, B.P. Chen, T.H. Chen, and T. Hai. 1996. ATF3 gene. Genomic organization, promoter, and regulation. *J Biol Chem*. 271:1695-701.

S6- Gately, D.P., and S.B. Howell. 1996. Paclitaxel activation of the GADD153 promoter through a cellular injury response element containing an essential Sp1 binding site. *J Biol Chem*. 271:20588-93.

S7- Yin, Y., Y.X. Liu, Y.J. Jin, E.J. Hall, and J.C. Barrett. 2003. PAC1 phosphatase is a transcription target of p53 in signalling apoptosis and growth suppression. *Nature*. 422:527-31.

S8- Wu, J., Y.J. Jin, G.M. Calaf, W.L. Huang, and Y. Yin. 2007. PAC1 is a direct transcription target of E2F-1 in apoptotic signaling. *Oncogene*. 26:6526-35.

S9- Lee, S.J., B.C. Jang, S.W. Lee, Y.I. Yang, S.I. Suh, Y.M. Park, S. Oh, J.G. Shin, S. Yao, L. Chen, and I.H. Choi. 2006. Interferon regulatory factor-1 is prerequisite to the constitutive expression and IFN-gamma-induced upregulation of B7-H1 (CD274). *FEBS Lett*. 580:755-62.

S10- Nakamura, M., T. Okura, Y. Kitami, and K. Hiwada. 2001. Nuclear factor 1 is a negative regulator of gadd153 gene expression in vascular smooth muscle cells. *Hypertension*. 37:419-24.

S11- Lawrence, M.C., K. McGlynn, B. Naziruddin, M.F. Levy, and M.H. Cobb. 2007. Differential regulation of CHOP-10/GADD153 gene expression by MAPK signaling in pancreatic beta-cells. *Proc Natl Acad Sci U S A*. 104:11518-25.

S12- Sauer, I., B. Schaljo, C. Vogl, I. Gattermeier, T. Kolbe, M. Muller, P.J. Blackshear, and P. Kovarik. 2006. Interferons limit inflammatory responses by induction of tristetraprolin. *Blood*. 107:4790-7.

S13- Inoue, D., S. Kido, and T. Matsumoto. 2004. Transcriptional induction of FosB/DeltaFosB gene by mechanical stress in osteoblasts. *J Biol Chem*. 279:49795-803.

S14- Cao, X., R. Mahendran, G.R. Guy, and Y.H. Tan. 1993. Detection and characterization of cellular EGR-1 binding to its recognition site. *J Biol Chem*. 268:16949-57.

Table 3 References
Contd…

S15- Castellanos, M.C., C. Munoz, M.C. Montoya, E. Lara-Pezzi, M. Lopez-Cabrera, and M.O. de Landazuri. 1997. Expression of the leukocyte early activation antigen CD69 is regulated by the transcription factor AP-1. *J Immunol*. 159:5463-73.

S16- Novak, E.M., M. Metzger, R. Chammas, M. da Costa, K. Dantas, C. Manabe, J. Pires, A.C. de Oliveira, and S.P. Bydlowski. 2003. Downregulation of TNF-alpha and VEGF expression by Sp1 decoy oligodeoxynucleotides in mouse melanoma tumor. *Gene Ther*. 10:1992-7.

S17- Shea, L.M., C. Beehler, M. Schwartz, R. Shenkar, R. Tuder, and E. Abraham. 1996. Hyperoxia activates NF-kappaB and increases TNF-alpha and IFN-gamma gene expression in mouse pulmonary lymphocytes. *J Immunol*. 157:3902-8.

S18- Rhoades, K.L., S.H. Golub, and J.S. Economou. 1992. The regulation of the human tumor necrosis factor alpha promoter region in macrophage, T cell, and B cell lines. *J Biol Chem*. 267:22102-7.

S19- Decker, E.L., N. Nehmann, E. Kampen, H. Eibel, P.F. Zipfel, and C. Skerka. 2003. Early growth response proteins (EGR) and nuclear factors of activated T cells (NFAT) form heterodimers and regulate proinflammatory cytokine gene expression. *Nucleic Acids Res*. 31:911-21.

S20- Fukai, T., C. Nishiyama, S. Kanada, N. Nakano, M. Hara, T. Tokura, S. Ikeda, H. Ogawa, and K. Okumura. 2009. Involvement of PU.1 in the transcriptional regulation of TNF-alpha. *Biochem Biophys Res Commun*. 388:102-6

Table - S5

These are the list of siRNA employed that were purchased from Qiagen.

Catalog No	Description
SI00945315	Mm_Cd69_1 HP siRNA(XM_132882)
SI01400847	Mm_Rgs1_1 HP siRNA (NM_015811)
SI00190155	Mm_Dusp2_1 HP siRNA (NM_010090
SI00910679	Mm_Axud1_1 HP siRNA (NM_153287
SI01478967	Mm_Zfp36_1 HP siRNA (NM_011756)
SI00207676	Mm_Tnf_1 HP siRNA (NM_013693)
SI00905863	Mm_Atf3_1 HP siRNA (NM_007498)
SI01373323	Mm_Pdcd1lg1_1 or CD274 HP siRNA(NM_021893)
SI00975667	Mm_Ddit3_1 HP siRNA (NM_007837)

Table - S6

Following are the list for the Phospho-specific antibodies from Cell Signaling technologies (Beverly, MA, USA) which were used:

Molecule Name	Phosphorylation site
1. Phospho-c-Raf	Ser338
2. Phospho-p38 MAPK	Thr180/Tyr182
3. Phospho-PKCdelta	Thr505
4. Phospho-p44/42 MAPK	(Thr202/Tyr204),
5. Phospho-Akt,	(Ser473)
6. Phospho-SAPK/JNK,	(Thr183/Tyr185)
7. Phospho-Bad,	(Ser112)
8. Phospho-PLCγ2,	(Tyr1217)
9. Phospho-MEK1/2,	(Ser217/221)
10. Phospho-GSK-3β,	(Ser9)
11. Phospho-SRC	(Tyr416)
11. Phospho-Lyn,	(Tyr507)
12. Phospho-PKA,	(Thr197)
13. Phospho-CaMKII,	(Thr286)
14. Phospho-Syk	(Tyr323)

Analysis for the mathematical model of BCR mediated signal initiation

The model at ground state

As already discussed that at ground state the value of $\alpha = 0$ and so the model (1) reduces to

$$\begin{aligned} \frac{dL_p}{dt} &= k_1 + k_2 L_p - d_1 L_p, \\ \frac{dS_m}{dt} &= A - k_3 S_m L_p, \\ \frac{dS_p}{dt} &= k_3 S_m L_p - d_2 S_p, \end{aligned} \tag{2}$$

Steady state analysis for the model at ground state

To find the steady state values of the system (2), we reduce the system of ordinary differential equation (2) to a system of algebraic equation by putting the left hand side of the equation equal to zero. i.e.,

$$\begin{aligned} 0 &= k_1 + k_2 L_p - d_1 L_p, \\ 0 &= A - k_3 S_m L_p, \\ 0 &= k_3 S_m L_p - d_2 S_p, \end{aligned} \tag{3}$$

Solving the system of equation (3) we get the steady state values. The system (2) has no boundary steady state. The positive steady state of the system (2) is given by $E^* \equiv (L_p^*, S_m^*, S_p^*)$, where

$$L_p^* = \frac{k_1}{d_1 - k_2}, \ S_m^* = \frac{A(d_1 - k_2)}{k_1 k_3}, \ S_p^* = \frac{A}{d_2}.$$

The positive steady state E^* exists if and only if $d_1 > k_2$.

The jacobian matrix of the system (2) around the positive steady state $E^* \equiv (L_p^*, S_m^*, S_p^*)$ is given by

$$J \equiv \begin{pmatrix} k_2 - d_1 & 0 & 0 \\ -k_3 S_m^* & -k_3 L_p^* & 0 \\ k_3 S_m^* & k_3 L_p^* & -d_2 \end{pmatrix}. \tag{4}$$

So, the eigenvalues corresponding to the jacobian matrix (4) are

$$k_2 - d_1, \ -k_3 L_p^*, \ -d_2. \tag{5}$$

Since all the eigenvalues will be negative if and only if $d_1 > k_2$, which is the existence condition for E^*. So whenever the positive steady state E^* exist it is stable.

Simulation of the Model

We first calculated the value of L_p for two different values of d_1 with other parameter values as in the Table 1 of Figure 7. Then the model system (1) is plotted with these two values of d_1, see Figure 7a. To plot Figure 7a we first integrated the system (2) with the initial value $L_p(0) = L_p^*$, $S_m(0) = S_m^*$, $S_p(0) = S_p^*$ up to $t = 5$. Since $E^* \equiv (L_p^*, S_m^*, S_p^*)$ is a stable steady state, so at the beginning a straight line is observed parallel to the x-axis up to $t = 5$. Again with the same L_p^*, S_m^*, S_p^* as the initial values we integrate the actual system (1) and plotted it after $t = 5$ in the same graph. Since our experiment is for finite time value, so we stopped our simulation after the first cycle. It is observed that the curve with higher peak value is for the lower basal value L_p^*, see Fig.1.

Additional Methods - IPA

Ingenuity Pathways Analysis (IPA)

Anti-IgM induced early gene expression regulatory module identified by IPA analysis that are represented in BCR NETWORK (14 focus genes in the dataset) containing activities related to cell death, cancer and hepatic system disease and the most significant canonical pathways represented in Figure 1C and D respectively. Upregulated genes are marked in red, whereas the downregulated ones are in green. The symbol code in the BCR NETWORK right describes the role of each of the nodes of the network.

Key for IPA network depicted in Figure 1

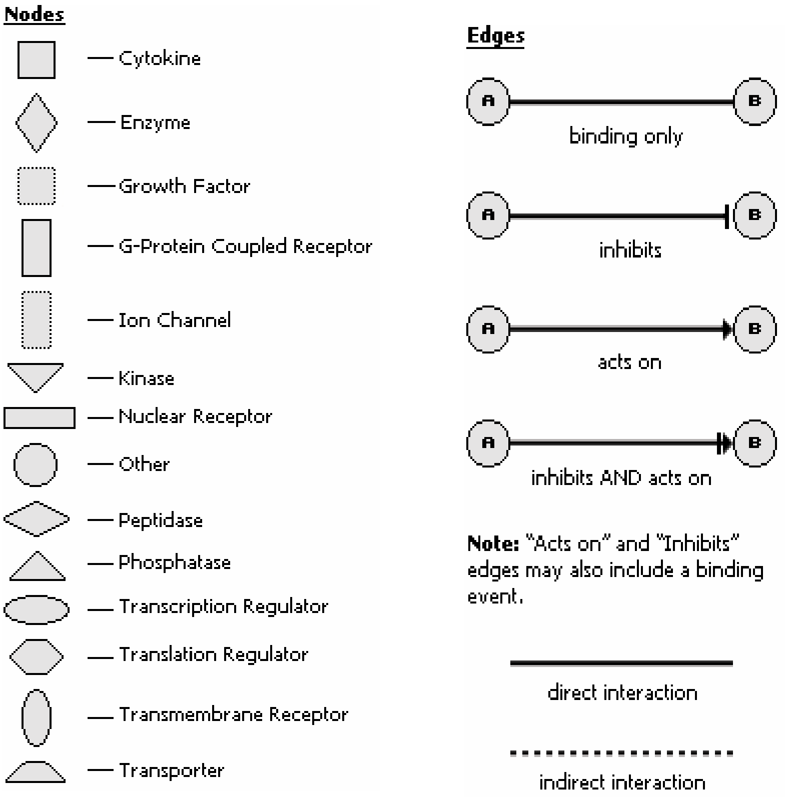

**EXPERIMENTAL DESIGN**
Definition of experimental and control groups
Number within each group
Assay carried out by core lab or investigator's lab?
Acknowledgement of authors' contributions
**SAMPLE**
Description
Volume/mass of sample processed
Microdissection or macrodissection
Processing procedure
If frozen - how and how quickly?
If fixed - with what, how quickly?
Sample storage conditions and duration (especially for FFPE samples)

Experimental design is provided in the material and method section. There were four groups namely normal unstimulated cells, anti-IgM treated cells, cells in presence of KN62 or SB203580. The samples were prepared in triplicates.

**NUCLEIC ACID EXTRACTION**
Procedure and/or instrumentation
Name of kit and details of any modifications
Source of additional reagents used
Details of DNase or RNAse treatment
Contamination assessment (DNA or RNA)
Nucleic acid quantification
Instrument and method
Purity (A260/A280)
Yield
RNA integrity method/instrument
RIN/RQI or Cq of 3' and 5' transcripts
Electrophoresis traces
Inhibition testing (Cq dilutions, spike or other)

Processing procedure was followed as per Qiagen kits guidelines. 5 x 10^6 cells were stimulated with anti-IgM for one hour with or with out treatment of the above mentioned inhibitors while untreated cells were used as control group. Stimulated CH1 cells were collected by centrifugation and resuspended in 1 ml of TRIZOL reagent. Complete lysis was ensured by repeated pipeting. The homogenized samples were incubated in room temperature for 5 mins, followed my addition of 200 µl of chloroform and mixed thoroughly. Samples were centrifuged at 13000 rpm for 15 mins at room temperature. The aqueous phase was transferred into a fresh tube and precipitated with 2.5 X volume of alcohol and kept overnight at ~80 degree centigrade. The RNA pellet was collected by centrifugation at 13000 rpm for 15 mins at room temperature.

The pellet was washed once with 75% alcohol, dried and responded in 50 µl of RNase-free water. Total RNA isolated was digested with RNase free DNase I prior to reverse transcription reaction. Estimation of relative transcript levels performed commercially from Labindia Life Sciences (Gurgaon, India) (http://www.labindia.com/index.htm).

REVERSE TRANSCRIPTION
Complete reaction conditions
Amount of RNA and reaction volume
Priming oligonucleotide (if using GSP) and concentration
Reverse transcriptase and concentration
Temperature and time
Manufacturer of reagents and catalogue numbers
Cqs with and without RT
Storage conditions of cDNA

TaqMan Assay Procedure

1. Use **500 ng** of total RNA per 100 µl RT reaction.
2. Prepare the RT master mix by scaling the volumes listed below to the desired number of RT Reactions (using **High Capacity cDNA RT kit using random hexamers**)

S.No.	Master Mix	1x Rxn in µl
1	10x RT buffer	10
2	25x dNTP	4
3	10x Random primer	10
4	RT Enz 50U/ µl	5
5	H_20	21
6	RNA	50
Total Vol		**100**

3. Mix gently and centrifuge to bring solution to the bottom of the tube.

Cycling Conditions -

Step Type	Time (Min)	Temperature (°C)
HOLD	10	25
HOLD	120	37
HOLD	∞	4

4. Set the reaction volume to 50 µl.
5. Load the reaction tube or plate into the thermal cycler.
6. Start the reverse transcription run.

qPCR TARGET INFORMATION
If multiplex, efficiency and LOD of each assay.
Sequence accession number
Location of amplicon
Amplicon length
In silico specificity screen (BLAST, etc)<br>Pseudogenes, retropseudogenes or other homologs?<br>Sequence alignment
Secondary structure analysis of amplicon
Location of each primer by exon or intron (if applicable)
What splice variants are targeted?

Sequence accession numbers are NM_007498.2, NM_153287.2, NM_021893.2, NM_007837.2, NM_010090.2, NM_015811.1and NM_011756.3.

qPCR OLIGONUCLEOTIDES
Primer sequences
RTPrimerDB Identification Number
Probe sequences
Location and identity of any modifications
Manufacturer of oligonucleotides
Purification method

Primer sequences and other details were not provided by the vendor.

qPCR PROTOCOL
Complete reaction conditions
Reaction volume and amount of cDNA/DNA
Primer, (probe), Mg++ and dNTP concentrations
Polymerase identity and concentration
Buffer/kit identity and manufacturer
Exact chemical constitution of the buffer
Additives (SYBR Green I, DMSO, etc.)
Manufacturer of plates/tubes and catalog number
Complete thermocycling parameters
Reaction setup (manual/robotic)
Manufacturer of qPCR instrument

PCR Amplification:

The recommended reaction volume is 10 µl. Prepare the plate so that each PCR reaction contains the components as listed in the following table.

S.No.	Component	Volume (5 µl Reaction)
1	TaqMan 2x MM	5.0 µl
2	AOD	0.5 µl
3	Sample (cDNA)	1.0 µl
4	Milli-Q	3.5 µl
TOTAL		**10.0 µl**

Cycling Conditions -

Step Type	Time (Min/Sec)	Temperature (°C)
HOLD	2min	50
HOLD	10min	95
40 cycles		
	15sec	95
	1min	60

Run Condition:

1. Run Module- Standard **7900 HT** Fast (Relative Quantification)
2. In the SDS software, open the plate document that corresponds to the reaction plate.
3. Load the reaction plate into the instrument.
4. Start the run.

**qPCR VALIDATION**
Evidence of optimisation (from gradients)
Specificity (gel, sequence, melt, or digest)
For SYBR Green I, Cq of the NTC
Standard curves with slope and y-intercept
PCR efficiency calculated from slope
Confidence interval for PCR efficiency or standard error
r2 of standard curve
Linear dynamic range
Cq variation at lower limit
Confidence intervals throughout range
Evidence for limit of detection
If multiplex, efficiency and LOD of each assay.

The complete validation was performed by the vendor as described here [1]. The final Ct values provided by the vendor are listed in Table S3.

DATA ANALYSIS
qPCR analysis program (source, version)
Cq method determination
Outlier identification and disposition
Results of NTCs
Justification of number and choice of reference genes
Description of normalisation method
Number and concordance of biological replicates
Number and stage (RT or qPCR) of technical replicates
Repeatability (intra-assay variation)
Reproducibility (inter-assay variation, %CV)
Power analysis
Statistical methods for result significance
Software (source, version)
Cq or raw data submission using RDML

qPCR analysis program (source, version): Biorad, Opticon 3
18S gene was used for reference gene which is ubiquitously expressed and its level does not change with experimental condition. Data normalization was performed using this reference gene along with three replicates. The results of analysis are listed in table S3. The entire normalization procedure was carried out by the vendor as described earlier [1].

References.

[1] Bookout, A. L.; Mangelsdorf, D. J., Quantitative real-time PCR protocol for analysis of nuclear receptor signaling pathways. *Nucl Recept Signal* **2003,** 1, e012.
